# Cannabidiol-Treated Ovariectomized Mice Show Improved Glucose, Energy, and Bone Metabolism With a Bloom in *Lactobacillus*

**DOI:** 10.3389/fphar.2022.900667

**Published:** 2022-06-21

**Authors:** Ke Sui, Kevin M. Tveter, Fiona G. Bawagan, Patricia Buckendahl, Savannah A. Martinez, Zehra H. Jaffri, Avery T. MacDonell, Yue Wu, Rocio M. Duran, Sue A. Shapses, Diana E. Roopchand

**Affiliations:** ^1^ Department of Food Science, NJ Institute for Food Nutrition and Health (Rutgers Center for Lipid Research and Center for Nutrition Microbiome and Health), Rutgers, The State University of New Jersey, New Brunswick, NJ, United States; ^2^ Molecular Imaging Center, Rutgers, The State University of New Jersey, New Brunswick, NJ, United States; ^3^ Department of Nutritional Sciences, NJ Institute for Food Nutrition and Health, Rutgers, The State University of New Jersey, and the Department of Medicine, Rutgers-RWJ Medical School, New Brunswick, NJ, United States

**Keywords:** cannabidiol, estrogen deficiency, inflammation, osteoporosis, gut microbiota, bile acids, lactobacillus

## Abstract

Loss of ovarian 17β-estradiol (E2) in postmenopause is associated with gut dysbiosis, inflammation, and increased risk of cardiometabolic disease and osteoporosis. The risk-benefit profile of hormone replacement therapy is not favorable in postmenopausal women therefore better treatment options are needed. Cannabidiol (CBD), a non-psychotropic phytocannabinoid extracted from hemp, has shown pharmacological activities suggesting it has therapeutic value for postmenopause, which can be modeled in ovariectomized (OVX) mice. We evaluated the efficacy of cannabidiol (25 mg/kg) administered perorally to OVX and sham surgery mice for 18 weeks. Compared to VEH-treated OVX mice, CBD-treated OVX mice had improved oral glucose tolerance, increased energy expenditure, improved whole body areal bone mineral density (aBMD) and bone mineral content as well as increased femoral bone volume fraction, trabecular thickness, and volumetric bone mineral density. Compared to VEH-treated OVX mice, CBD-treated OVX mice had increased relative abundance of fecal *Lactobacillus* species and several gene expression changes in the intestine and femur consistent with reduced inflammation and less bone resorption. These data provide preclinical evidence supporting further investigation of CBD as a therapeutic for postmenopause-related disorders.

## 1 Introduction

Decline in the production of ovarian 17β-estradiol (E2) during menopause and postmenopause places women at greater risk for weight gain, cardiometabolic disease, osteoporosis, and gastrointestinal disorders (Monteleone et al., 2018). Loss of ovarian E2 leads to systemic physiological changes including an altered gut microbiota (Flores et al., 2012; Choi et al., 2017; Chen et al., 2021) and dysregulation of metabolic functions (Mauvais-Jarvis et al., 2013). In obese postmenopausal women, over one hundred bacterial taxa were correlated with markers of metabolic disease such as inflammation, lipid metabolism, and insulin resistance (Brahe et al., 2015). In humans and rodent models, E2 decline was further associated with decreased expression of tight junction proteins in the intestinal epithelium leading to impaired barrier function (Li et al., 2016; Collins et al., 2017; Shieh et al., 2020; Yu M. et al., 2021). A leaky gut barrier leads to metabolic endotoxemia where passage of proinflammatory bacterial components, such as lipopolysaccharide (LPS), into the lamina propria causes immune cell activation and production of proinflammatory cytokines that enter circulation and other tissue (Li et al., 2016; Collins et al., 2017; Shieh et al., 2020; Yu M. et al., 2021). The resulting state of chronic low-grade inflammation contributes to metabolic disease and bone loss (Mauvais-Jarvis et al., 2013; Li et al., 2016; Shieh et al., 2020). In addition to gut bacterial changes, E2 deficiency has been found to alter the levels of bacterial metabolites, including short chain fatty acids (SCFAs) and bile acids (Chen et al., 2021).

Estrogens regulate bone remodeling, energy metabolism, and gut bacteria (Mauvais-Jarvis et al., 2013; Shieh et al., 2020). E2 decline during menopause and postmenopause stimulates bone resorption leading to a period of rapid bone loss that can progress to osteoporosis and increased risk of fracture (Monteleone et al., 2018). Bone fractures are most common in the hip and vertebrae and can have serious consequences in the elderly. Hip fractures are associated with up to 20% increased mortality in the first year and 20–50% of survivors require long term nursing home care (Johnston and Dagar, 2020). Postmenopausal osteoporosis is characterized by increased osteoclast-induced bone resorption and decreased osteoblast-driven bone formation (Udagawa et al., 2021). The RANK/RANKL/OPG signaling pathway regulates the balance of bone resorption and bone formation (Udagawa et al., 2021). Osteoclastogenesis and bone resorption occurs when receptor activator of nuclear factor κB (RANK) on osteoclast cell membranes interacts with membrane-associated RANK-Ligand (RANKL) expressed on osteoblasts, osteocytes, and B and T lymphocytes (Eghbali-Fatourechi et al., 2003; Taxel et al., 2008). E2 suppresses bone resorption by stimulating osteoblasts to produce osteoprotegerin (OPG), a secretory protein that binds RANKL, preventing its interaction with RANK (Udagawa et al., 2021). In postmenopause, E2 deficiency leads to expansion of gut-derived immune cells that migrate to bone to produce proinflammatory cytokines and RANKL, which enhances osteoclastogenesis and bone loss (Yu M. et al., 2021).

If commenced within first 5 years of postmenopause, a personalized regimen of hormone replacement therapy (HRT) can help alleviate vasomotor symptoms and reduce risk of bone fracture due to osteoporosis (Grossman et al., 2017). The lowest dose of HRT for no more than 5 years is generally recommended to minimize risks of chronic disease (Grossman et al., 2017). HRT is not advised for women over 65 years and/or 10 years postmenopausal due to elevated risks of cancer, heart disease, stroke, and dementia (Grossman et al., 2017). Bisphosphonates and selective estrogen receptor modulators are also prescribed for postmenopausal osteoporosis prevention or treatment each with their own risk-benefit profiles (Khosla and Hofbauer, 2017). Other therapeutic options with fewer safety concerns are needed for prevention of chronic disease in this underserved demographic.

CBD, a non-psychotropic phytocannabinoid derived from the industrial hemp plant (*Cannabis sativa* L.), has displayed diverse pharmacological activities relevant to postmenopause including antioxidant and anti-inflammatory activities (De Filippis et al., 2011; Atalay et al., 2019; Nichols and Kaplan, 2020), improved gut barrier (Cocetta et al., 2021), protection from collagen-induced arthritis (Malfait et al., 2000), and reduced bone loss (Napimoga et al., 2009; Li D. et al., 2017; Raphael-Mizrahi and Gabet, 2020). CBD (Epidiolex^®^) is currently FDA-approved for treatment of epilepsy-related disorders in children and adults with a favorable safety profile (Bergamaschi et al., 2011; Iffland and Grotenhermen, 2017; Larsen and Shahinas, 2020; Gaston et al., 2021). CBD is highly lipophilic and reported to have relatively low bioavailability (∼6%) if consumed during fasting but can increase 4-fold if consumed with a high fat meal (Millar et al., 2018; Perucca and Bialer, 2020). Over 65 CBD targets or receptors have been identified throughout the body, including intestine and bone (Britch et al., 2021). Endocannabinoid receptors type-1 (CB1) and type-2 (CB2), transient receptor potential vanilloid (TRPV) receptors, and G protein-coupled receptor (GPR)55 are CBD receptors known to be present in osteoclasts and osteoblasts (Idris and Ralston, 2010). In the present study we investigated the effects of CBD on several postmenopause-related symptoms using the classical ovariectomized (OVX) mouse model along with sham surgery (SS) controls and show that CBD can modulate a gut-bone axis to attenuate chronic disease symptoms resulting from E2 deficiency.

## 2 Materials and Methods

### 2.1 Animals and Treatment

Animal studies were conducted according to protocols approved by Rutgers institutional animal care and use committee. Female wild-type C57BL/6J mice (*n* = 40) aged 8 weeks were purchased from Jackson Laboratory (Bar Harbor, ME) and housed (5 mice/cage) under controlled conditions (24 ± 1°C, 12 h light/dark cycle) with *ad libitum* access to chow and water. Mice were OVX (*n* = 20) or underwent sham-surgery (SS, *n* = 20) at 12-weeks of age and were individually housed after surgery. After a 10-days recovery period, mice were switched from chow to a purified diet containing 10 kcal% from fat (D12450J, Research Diets, New Brunswick, NJ) and water. Five days after the diet transition, OVX and SS groups were equally and randomly subdivided into CBD and vehicle (VEH) treatment groups (*n* = 10 mice/group). CBD isolate (96–99% purity) was purchased from Bluebird Botanical (Louisville, CO). Starting at 14 weeks of age, for 18 weeks (5 days per week excluding weekends) mice were perorally dosed with VEH (sesame oil) or CBD (25 mg/kg, dissolved in sesame oil, 10 mg/ml) where the calculated volume of VEH or CBD was mixed in ∼100 mg of powdered peanut butter and once presented to mouse the entire dose was consumed within 1 min. Two mice (one from each OVX group) were lost during metabolic testing. After 18-weeks, mice (aged 32 weeks) were euthanized by CO_2_ inhalation and blood was collected immediately by cardiac puncture, followed by isolation of serum. Uterus and liver were weighed. Ileum and colon segments were dissected as previously described (Tveter et al., 2020; Mezhibovsky et al., 2021) and then flushed with ice-cold, sterile PBS (pH 7.4) to collect luminal contents for targeted metabolomics. Samples were placed in cryogenic tubes, snap frozen in liquid nitrogen, and stored at −80°C until analysis. Animal carcasses were stored frozen at −80°C until bone analyses.

### 2.2 Metabolic Tests

Body weight and food intake was measured weekly. Body composition was measured using an EchoMRI body composition analyzer (EMR-129, Echo Medical Systems, Houston, TX) at 0, 4, 7, 10, 14, and 18 weeks post-treatment. After 9 weeks of treatment, an oral glucose tolerance test was performed as previously described (Tveter et al., 2020; Mezhibovsky et al., 2021). During the 9th and 10th week post-treatment, indirect calorimetry was performed on 9 mice per group using a Comprehensive Laboratory Animal Monitoring System with temperature and light control enclosure (CLAMS, Columbus Instruments, Columbus, Ohio) as previously described (Mezhibovsky et al., 2021).

### 2.3 Bone Densitometry

Total body areal bone mineral density (aBMD) and bone mineral content (BMC) was measured using dual-energy X-ray absorptiometry (DEXA, GE-Lunar PIXImus mouse densitometer software version 2.10.41) as previously described (Wang et al., 2016). Frozen carcasses were used for whole body scanning. Immediately after each scan (within 2–3 min), the frozen headless carcass was placed on dry ice. Individual right-side femur, tibia, humerus, and lumbar spine (L1-L5) were dissected to remove surrounding tissues and scanned by DEXA. After scanning the individual right-side bones were wrapped with phosphate buffered saline (PBS) soaked gauze and stored at −20°C. The left femur was dissected, placed in a microfuge tube, and stored frozen at −80°C for RNA extraction.

### 2.4 Bone Microcomputed Tomography (microCT)

Right femur samples (stored at −20°C) were placed on dry ice and transported to Rutgers Imaging Center for microCT analysis. Tubes containing femurs were placed into room temperature water to thaw, wrapped securely with parafilm to prevent drying, and inserted into a 5 mm polystyrene tube to be scanned using a microcomputed tomography system (SkyScan 1,272; Bruker Corporation, Kontich, Belgium). Settings were 80 kV, 124 μA, image voxel size 6.0 µm isotropically, and camera image settings 1,092 × 1,632 pixels with 0.1 mm Al filter, averaging 3 frames per rotation step of 0.4°. The NRecon was used to reconstruct the scanned images of femoral microstructure and analyzed with SkyScan CT Analyzer (Bruker microCT version 1.18.1.0). Geometric trabecular volumetric BMD (vBMD) and cortical tissue mineral density (TMD) were analyzed and calculated as mg/mL against calibrated phantoms of 250 and 750 mg hydroxyapatite/cc as the standards. Femoral 3D images were generated using CTvol (Bruker microCT version 3.3.0) (Bouxsein et al., 2010).

### 2.5 Quantitative Polymerase Chain Reaction

RNA from ileum and colon tissues (30–35 mg) were extracted as previously described (Tveter et al., 2020; Mezhibovsky et al., 2021). Each femur bone sample (stored at −80°C) was ground to powder with a mortar and pestle (pre-chilled in −20°C freezer) containing liquid nitrogen. Each powdered femur sample was transferred to a microfuge tube and 900 µL of Qiazol was added for total RNA extraction according to kit instructions (RNeasy Plus universal kit, QIAGEN). RNA concentrations were quantified by Nanodrop (Thermo Fisher Scientific Inc.). cDNA was synthesized from 5 µg RNA and RT-qPCR was performed (with technical duplicates) on a thermocycler (Quantstudio 3, Thermo Fisher Scientific Inc.) using Taqman™ Fast universal PCR master mix using conditions: 20 s at 95°C followed by 40 cycles of 95°C for 1 s (denaturation) and 60°C for 20 s (annealing and extension). TaqMan™ assay primers (Life Technologies) used are summarized in Supplementary Table S2. *Hmbs* and *Gapdh* were used as housekeeping genes for intestinal and femoral tissues, respectively.

### 2.6 Targeted Metabolomics

Previously described (Tveter et al., 2020) methods with modifications described in supplementary methods were used for analysis of serum and ileal content bile acids (BAs). Cecal short chain fatty acids (SCFA) were analyzed by GC-MS using previously described methods (García-Villalba et al., 2012).

### 2.7 Gut Microbiota Analysis

gDNA was extracted from week 18 fecal samples (*n* = 28, 7 per group) using DNeasy PowerSoil Pro Kit (QIAGEN). Library preparation and paired-end sequencing of 16S rRNA V3-V4 amplicons (2 × 250 bp configuration) was performed by Azenta LifeSciences (Piscataway, NJ) using an Illumina^®^ MiSeq instrument.

To confirm the increase in *Lactobacillus* sp., qPCR was performed using primers (Lac_groEL_F: 5′-GCYGGTGCWAACCCNGTTGG-3′ and Lac_groEL_R: 5′-AANGTNCCVCGVATCTTGTT-3′) targeting the previously described *Lactobacillus*-specific *groEL* region (Yasrebi et al., 2017) using conditions: 5 min at 95°C, 40 cycles of 30 s at 95°C, 30 s at 58°C, 60 s at 72°C, ending with 72°C for 7 min. A melt curve stage of 15 s at 95°C, 60 s at 60°C, and 15 s at 95°C ended the run, a single peak confirmed amplification of a single PCR product.

### 2.8 Statistics

Data were analyzed using GraphPad Prism 8 software (GraphPad Software, Inc., La Jolla, CA, United States). The ROUT test was used to detect and remove outliers, if any. Data were tested for normality and variance prior to using parametric or non-parametric tests. For tissue gene expression qPCR data, difference between groups (mean ± SD) was determined by two-way ANOVA followed by Holm-Sidak test (*p* < 0.05 considered significantly different) to compare groups with difference of one factor only, i.e., VEH or CBD treatment within surgery group (VEH vs. CBD for SS or OVX) or comparing effect of surgery within treatment (SS vs. OVX for VEH or CBD treatment). Difference between all four groups for mouse phenotypes, bile acid, and gut bacteria qPCR were determined by two-way ANOVA followed by the original FDR method of Benjamini and Hochberg post-hoc test (q < 0.05 considered significantly different). Exact q and/or *p* values are indicated for notable trends. For time series data, 2-way ANOVA and posthoc test was performed on the last time point and repeated on earlier time points until differences were no longer significant. For beta diversity metrics analysis of variance was determined using ADONIS and 10,000X permutation analysis in the vegan package within R Studio v.4.1.2 (R Studio Software, Boston, Massachusetts, United States). To determine difference between gut bacteria at the phylum and genus levels (which were a mix of normally and non-normally distributed data) and in cases where bile acids were non-normally distributed, the non-parametric Kruskal–Wallis test was used followed by the Benjamini–Hochberg post-hoc test.

## 3 Results

### 3.1 Cannabidiol Improved Glucose Tolerance and Energy Metabolism in Ovariectomized Mice

**FIGURE 1:**
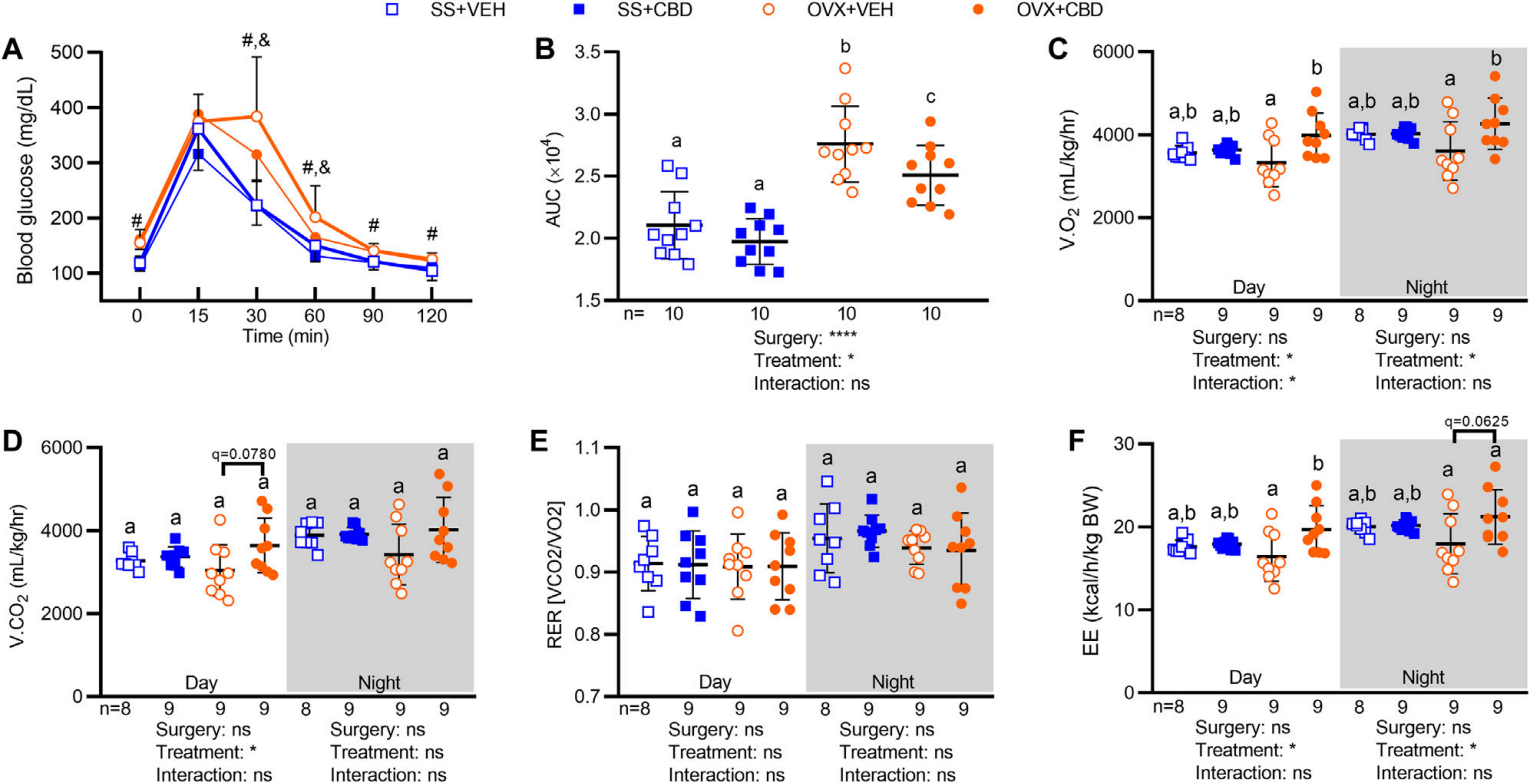
CBD increased oral glucose tolerance, O2 consumption, and energy expenditure in OVX mice. **(A)** After 9 weeks of CBD or VEH treatment, blood glucose readings were taken at 0, 15, 30, 60, 90 and 120 min after oral glucose challenge (2 mg/kg), *n* = 10 mice/group. **(B)** Oral glucose tolerance data in **(A)** represented as area under the blood glucose curves (AUC). Indirect calorimetry performed between 9 and 10 weeks on 9 mice/group showing **(C)** volume of oxygen consumed, V.O2, **(D)** volume of carbon dioxide produced, V.CO2 **(E)** respiratory exchange ratio, RER, and **(F)** energy expenditure, EE (as kcals expended per hour normalized to body weight in kg). Data are mean ± SD. The ROUT test was performed to detect outliers. 2-way ANOVA was performed followed by the Benjamini–Hochberg post-hoc test with FDR adjustment, q < 0.05. The 2-way ANOVA results for panel A are summarized in Supplementary Table S1. In panel A, pound (#) sign denotes significant difference between SS groups and OVX groups and ampersand (&) sign denotes significant difference between OVX+VEH and OVX+CBD groups, as determined by Benjamini–Hochberg posthoc test. Two-way ANOVA results are indicated under graphs **(B–F)**. Different letters **(a–c)** indicate significant differences determined by the Benjamini–Hochberg post-hoc test with FDR-adjusted *p* values, q < 0.05.

LC-MS quantification showed that CBD concentrations were 10-fold higher in ileal content (0.01—0.03 μg/ml) than in serum (0.002 μg/ml, Table 1). OVX mice treated with CBD for 9 weeks showed improved oral glucose tolerance (OGT) compared to VEH-treated OVX mice, although both OVX groups had less efficient glucose clearance compared to ovary-intact VEH and CBD-treated SS groups (Figures 1A,B). CBD did not alter food intake or body composition over the 18-weeks treatment period; however, compared to SS groups, OVX mice had decreased percent lean mass and increased body weight, percent fat mass, and liver weights (Supplementary Figures S1A–G). Relevant differences were not observed between groups for liver weights normalized to body weights (Supplementary Figure S1H). CBD did not affect ovariectomy-induced uterine weight decrease and atrophy or the decrease in uterine/body weight ratio, phenotypes confirming loss of E2 in OVX mice as compared to the SS group (Supplementary Figures S1I–J) (Yasrebi et al., 2017). Energy metabolism was assessed by indirect calorimetry after 9–10 weeks of CBD treatment. CBD-treated OVX mice had increased O2 consumption during day and night phases (Figure 1C) and there was a trend of increased CO2 production during the day (Figure 1D). Respiratory exchange ratio (RER) was not affected by CBD or ovariectomy (Figure 1E). Compared to VEH-treated OVX mice, energy expenditure (EE) in CBD-treated OVX mice was increased during the day and trended higher during the night phase while OVX- and VEH-treated SS groups had similar EE (Figure 1F). Compared to SS groups, spontaneous physical activity was lower in the OVX groups for Y-plane activity during the night phase (Supplementary Figures S2C,D).

**TABLE 1:** Bile acid concentrations in ileal content and serum.

Group		SS + VEH	SS + CBD	OVX + VEH	OVX + CBD	SS + VEH	SS + CBD	OVX + VEH	OVX + CBD
BA	Type	Ileum BA concentration (μg/mg Ileal Content)				Serum BA concentration (μg/mL Serum)			
**αMCA**	1°	28 ± 27	32 ± 24	54 ± 38	89 ± 102	0.07 ± 0.05	0.04 ± 0.03	0.06 ± 0.06	0.06 ± 0.049
**βMCA**	1°	53 ± 51	109 ± 98	233 ± 264	137 ± 146	0.8 ± 0.7	0.3 ± 0.3	0.34 ± 0.3	0.25 ± 0.2
**CA**	1°	900 ± 835	1,131 ± 795	1,136 ± 828	2,122 ± 1961	0.5 ± 0.4	0.2 ± 0.13	0.23 ± 0.2	0.53 ± 0.5
**CDCA**	1°	13 ± 13.6	8.2 ± 8.1	6.6 ± 7.7	8.1 ± 7.1	0.006 ± 0.004^b^	0.003 ± 0.003^a,b^	0.005 ± 0.003^a,b^	0.002 ± 0.002^a^
**GCA**	1°	17 ± 13^a^	15 ± 6^a^	40 ± 18^b^	21 ± 11^a^	0.003 ± 0.004	0.002 ± 0.002	0.003 ± 0.004	N.D
**GCDCA**	1°	0.05 ± 0.1^a^	0.04 ± 0.03^a^	0.13 ± 0.1^b^	0.1 ± 0.1^a,b^	0.0004 ± 0.0006	0.0004 ± 0.0005	0.0002 ± 0.0005	0.0002 ± 0.0004
**GUDCA**	1°	0.9 ± 0.7^a^	0.8 ± 0.4^a^	4 ± 4.4^b^	1.5 ± 1.1^a,b^	0.014 ± 0.005	0.013 ± 0.003	0.012 ± 0.002	0.012 ± 0.003
**TCA**	1°	49 ± 15^a,b^	42 ± 23^a,b^	74 ± 35^b^	40 ± 16^a^	0.1 ± 0.03	0.1 ± 0.06	0.13 ± 0.14	0.1 ± 0.07
**TCDCA**	1°	110 ± 99	54 ± 42	29 ± 17	43 ± 27	0.006 ± 0.01	0.003 ± 0.003	0.007 ± 0.007	0.005 ± 0.003
**TUDCA**	1°	673 ± 418	737 ± 374	1,057 ± 783	795 ± 376	0.2 ± 0.6^b^	0.1 ± 0.1^a^	0.05 ± 0.03^a^	0.1 ± 0.05^a,b^
**TαMCA**	1°	147 ± 59^a,b^	105 ± 22^a^	260 ± 199^b^	157 ± 71^a,b^	0.03 ± 0.01^b^	0.01 ± 0.01^a^	0.03 ± 0.02^b^	0.01 ± 0.010^a^
**TβMCA**	1°	294 ± 105^a,b^	211 ± 95^a^	490 ± 318^b^	299 ± 121^a,b^	0.03 ± 0.02	0.02 ± 0.02	0.04 ± 0.04	0.02 ± 0.02
**UDCA**	1°	10.2 ± 10	6.7 ± 6.5	4.2 ± 5	5.7 ± 5	0.02 ± 0.12^b^	0.008 ± 0.006^a^	0.011 ± 0.006^a^	0.005 ± 0.003^a^
**DCA**	2°	24 ± 34	20 ± 18	12 ± 13	16 ± 12	0.11 ± 0.06^b^	0.05 ± 0.02^a^	0.07 ± 0.05^a,b^	0.03 ± 0.014^a^
**GDCA**	2°	0.1 ± 0.1	0.07 ± 0.1	0.02 ± 0.03	0.05 ± 0.02	0.0004 ± 0.004	0.0003 ± 0.003	0.0005 ± 0.006	0.0003 ± 0.0004
**GHCA**	2°	N.D	N.D	N.D	N.D.	0.0003 ± 0.0008	0.0004 ± 0.0008	0.0004 ± 0.0009	N.D
**GLCA**	2°	0.015 ± 0.04	0.009 ± 0.027	N.D	N.D	0.007 ± 0.01	0.013 ± 0.02	0.01 ± 0.01	0.004 ± 0.005
**HCA**	2°	1 ± 0.6^a^	1.8 ± 1.1^a^	4 ± 3^b^	1.5 ± 0.6^a^	0.02 ± 0.01	0.02 ± 0.01	0.024 ± 0.01	0.023 ± 0.01
**HDCA**	2°	19 ± 14	11 ± 7	24 ± 29	12 ± 10	0.03 ± 0.01	0.03 ± 0.01	0.04 ± 0.01	0.03 ± 0.001
**isoDCA**	2°	N.D	N.D	N.D	N.D	0.0001 ± 0.0001	N.D	4E-05 ± 6E-05	0.0001 ± 9E-05
**MDCA**	2°	2.7 ± 2.6	3 ± 2.5	14 ± 17	9 ± 10	0.014 ± 0.01^b^	0.005 ± 0.005^a^	0.007 ± 0.003^a^	0.008 ± 0.004^a,b^
**NCA**	2°	7.5 ± 6	2.4 ± 3	2 ± 3	6 ± 5.6	0.02 ± 0.03	0.004 ± 0.005	0.003 ± 0.004	0.005 ± 0.01
**TDCA**	2°	75 ± 53	44 ± 40	25 ± 11	43 ± 37	0.034 ± 0.01^a^	0.02 ± 0.01^b^	0.015 ± 0.01^b^	0.014 ± 0.01^b^
**THDCA**	2°	70 ± 41^a^	70 ± 41^a^	183 ± 118^b^	105 ± 35^a,b^	0.4 ± 0.5	0.3 ± 0.2	0.4 ± 0.2	0.3 ± 0.2
**TLCA**	2°	2 ± 1^b^	0.8 ± 0.6^a^	0.9 ± 0.6^a,b^	0.6 ± 0.2^a^	N.D	N.D	0.001 ± 0.003	N.D
**TωMCA**	2°	382 ± 145	255 ± 100	292 ± 178	234 ± 103	0.08 ± 0.02^a,b^	0.06 ± 0.03^a,b^	0.1 ± 0.03^b^	0.05 ± 0.02^a^
**ωMCA**	2°	17 ± 17^a^	30 ± 30^a^	148 ± 149^b^	31 ± 36^a^	0.587 ± 0.42	0.35 ± 0.19	0.54 ± 0.32	0.39 ± 0.3
**Unconjugated BAs**		867 ± 697	1,514 ± 1,191	1,376 ± 851	2,466 ± 2,267	2.2 ± 1.4	0.9 ± 0.3	1.5 ± 1	1.3 ± 1
**Conjugated BAs**		1851 ± 641	1,614 ± 721	2,228 ± 1932	1899 ± 479	0.7 ± 0.2	0.6 ± 0.3	0.8 ± 0.5	0.65 ± 0.3
**G-conjugated**		18 ± 14^a^	16 ± 6^a^	43 ± 20^b^	22 ± 12^a^	0.02 ± 0.01	0.05 ± 0.04	0.02 ± 0.01	0.01 ± 0.005
**T-conjugated**		1833 ± 631	1,600 ± 716	2,490 ± 1794	1876 ± 477	0.7 ± 0.2	0.5 ± 0.3	0.8 ± 0.5	0.6 ± 0.3
**PBAs**		2,391 ± 1,178	2,286 ± 681	2,476 ± 1,418	3,778 ± 2,299	2 ± 1.3^b^	0.7 ± 0.4^a^	0.9 ± 0.5^a,b^	1.1 ± 0.8^a,b^
**SBAs**		678 ± 226	472 ± 123	970 ± 675	460 ± 151	1.35 ± 0.9	0.82 ± 0.3	1.33 ± 0.4	0.81 ± 0.43
**Total BAs**		3,060 ± 1,284	2,839 ± 881	7,149 ± 7,961	4,356 ± 2,473	3.3 ± 2.1^b^	1.7 ± 1.0^a^	2.4 ± 0.7^a,b^	1.9 ± 1.2^a,b^
**CBD (μg/ml)**		N.D	0.03 ± 0.02	N.D	0.01 ± 0.007	N.D	0.002 ± 0.001	N.D	0.002 ± 0.001

Targeted LC-MS, analysis of BAs in ileum content and serum from sham surgery (SS) and ovariectomized (OVX) mice treated with vehicle (VEH) or cannabidiol (CBD, 25 mg/kg body wt.). Each sample was injected in duplicate using negative and positive ionization. To prevent residual BAs, from eluting into the subsequent sample, a 20-min column wash and calibration was performed between each sample injection. Individual BA, concentrations were determined via external standard curves with internal standard calibration. Concentrations are reported as mean ± S.D., BAs not detected (N.D.) were assigned value of 0 for statistics. BAs, conjugated with either Taurine (T) or *Glycine* (G) were grouped as conjugated BAs, T-conjugated, or G-conjugated while unconjugated BAs, refer to BAs that do not contain T or G in their structure. Primary BAs (PBAs) refer to BAs, produced in host liver and secondary BAs (SBAs) refer to PBAs, that are microbially modified. Significant difference was assessed using 2-way ANOVA, followed by the Benjamini–Hochberg post-hoc test with false-discovery rate adjusted *p* values. For comparisons between groups where BA, levels were undetectable (N.D.) a Kruskal Wallis test followed by the Benjamini–Hochberg post-hoc test with false-discovery rate adjusted *p* values was applied. Different letters denote significant difference (q < 0.05) when comparing all groups. BA, concentrations without any letters or asterisks were not significantly different between groups.

### 3.2 Cannabidiol Improved Markers of Intestinal Inflammation and Gut Barrier

**FIGURE 2:**
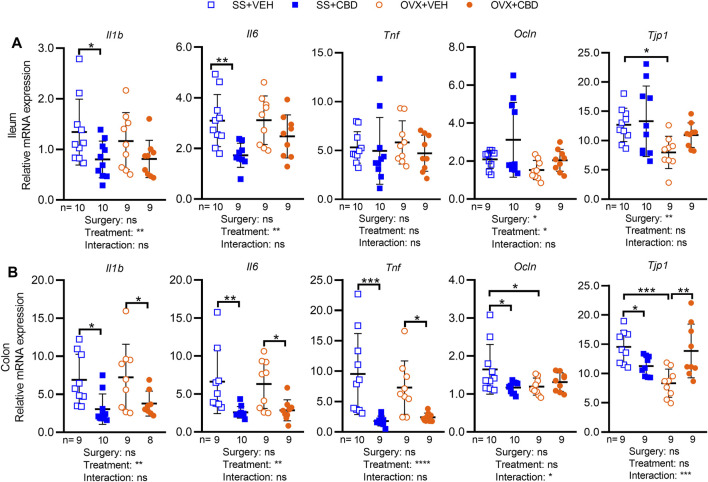
CBD treatment decreased markers of intestinal inflammation. Gene expression levels of inflammatory markers (IL-1β, IL-6 and TNFα) and tight junction proteins (occludin and ZO-1) in **(A)** ileum and **(B)** colon collected from *n* = 9 mice in OVX groups and *n* = 10 mice in SS groups. All data are mean ± SD. The ROUT test was performed to detect outliers. Significant differences between groups were determined by two-way ANOVA followed by Holm-Sidak post-hoc test to isolate differences due to surgery or treatment, *p < 0.05, **p < 0.01, ***p < 0.001.

We investigated the impact of CBD on intestinal markers of gut inflammation, gut barrier integrity, and nutrient metabolism. Compared to VEH-treated OVX mice, CBD-treated OVX mice showed a trend of lower *Il1b* (*p* = 0.146) and *Il6* (*p* = 0.124) mRNA levels in ileal tissues (Figure 2A) and significantly lower *Il1b, Il6* and *Tnf* in colon tissues (Figure 2B). CBD-treated SS mice had lower ileal expression of *Il1b* and *Il6* (Figure 2A) and decreased colonic expression of *Il1b, Il6* and *Tnf* (Figure 2B). Compared to the VEH-treated OVX group, CBD-treated OVX mice had increased colonic mRNA levels of *Tjp1*, encoding tight junction protein 1 (i.e., zonula occludens-1, ZO-1) while *Ocln*, encoding occludin was unchanged (Figure 2B); expression of *Ocln* and *Tjp1* were unchanged in ileum (Figure 2A). Compared to the VEH-treated SS group, the CBD-treated SS group showed decreased *Ocln* and *Tjp1* in colon tissues (Figure 2B), an unexpected result given the concomitant increase in ileal *Tjp1* (Figure 2A) and reduced mRNA levels of *Il1b* and *Il6* in the ileum and colon tissues of CBD-treated SS mice (Figures 2A,B). These data indicated that CBD treatment reduced intestinal inflammation in SS and OVX groups, possibly due to improved gut barrier integrity in the OVX group.

To investigate explanations for the improved glucose tolerance, we further examined ileal gene expression of nutrient signaling peptides, glucose transporters, and bile acid receptors. *Gcg* encodes preproglucagon, which is cleaved by PC1/3 protease (*Pcsk1*) to yield the incretin peptide Glp1 and Glp2, which regulates gut barrier, intestinal hexose transport, and bone metabolism (Schiellerup et al., 2019). *Gcg* expression was unaffected by CBD or OVX (Supplementary Figure S3A). *Pcsk1* expression was similar between VEH- and CBD-treated OVX groups; however, reduced *Pcsk1* expression was observed in the CBD-treated SS and VEH-treated OVX groups when compared to the VEH-treated SS group (Supplementary Figure S3A) suggesting reduced production of GLP1 and GLP2 peptides. CBD treatment did not alter ileal gene expression of carbohydrate transporters SGLT1 and GLUT2, encoded by *Slc5a1* and *Slc2a2*, respectively, suggesting improved glucose tolerance was not a consequence of altered carbohydrate absorption (Supplementary Figure S3A). Membrane bound Takeda G-protein coupled receptor 5 (TGR5), encoded by *Gpbar1*, and nuclear transcription factor farnesoid-X receptor (FXR), encoded by *Nr1h4*, are bile acid receptors regulating glucose metabolism (Pathak et al., 2017). Significant differences were not detected in ileal mRNA levels of *Nr1h4, Gpbar1* or *Fabp6*, a target gene of activated FXR (Supplementary Figure S3A). These data suggest that improved glucose tolerance in CBD-treated OVX mice was independent of changes to intestinal carbohydrate absorption, incretin production, or BA receptor expression.

### 3.3 Cannabidiol Attenuated Ovariectomized-Induced Osteoporosis and Suppressed Femoral Markers of Bone Resorption and Inflammation

**FIGURE 3:**
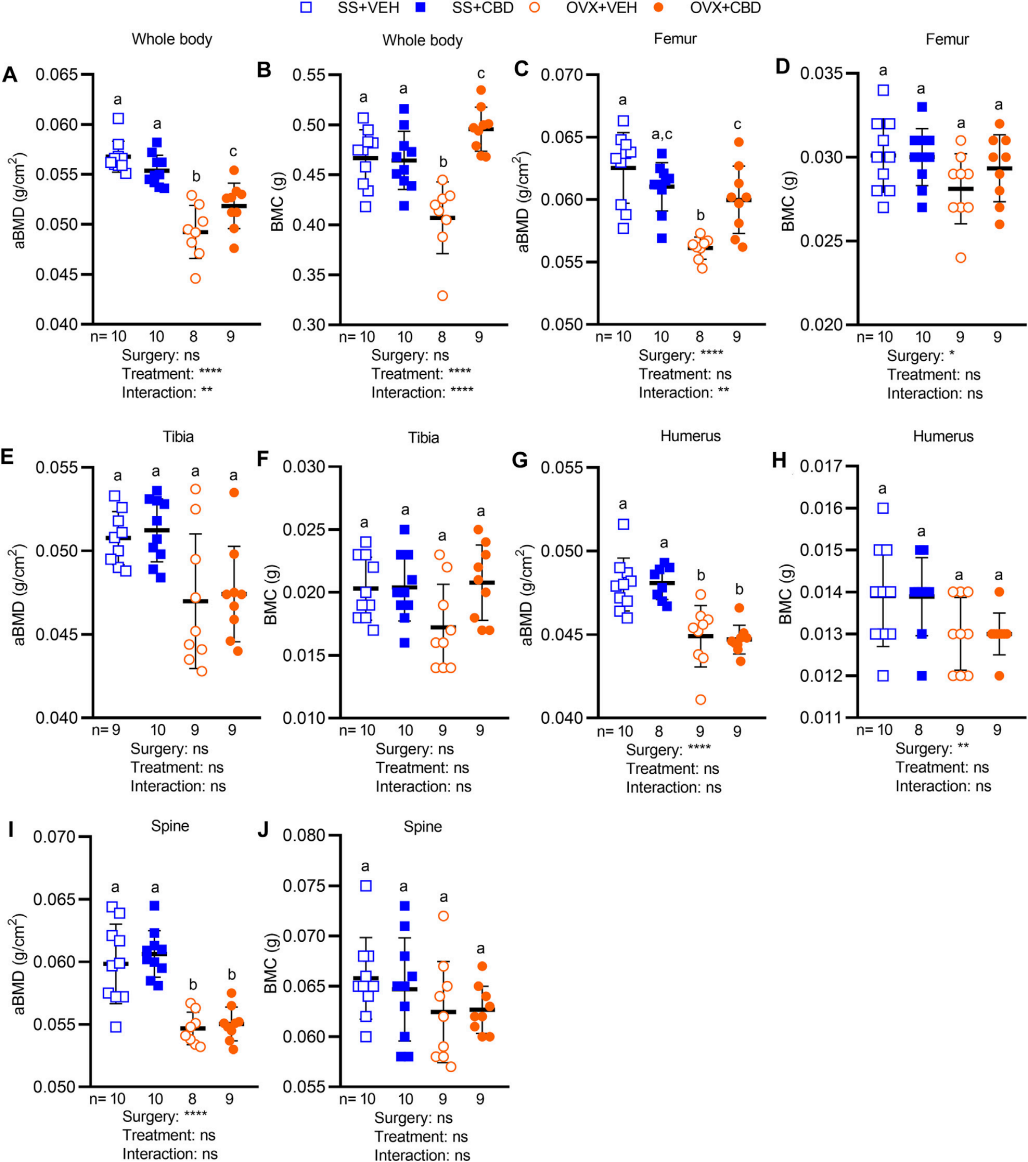
CBD alleviated bone loss in OVX mice. Areal bone mineral density (aBMD) and bone mineral content (BMC) of **(A,B)** whole body and individual bone samples of **(C,D)** femur, **(E,F)** tibia **(G,H)** humerus, and **(I,J)** spine as measured by DEXA. Data are presented as mean ± SD and were collected from 9 mice in OVX groups and 10 mice in SS groups, except for humerus data in the SS + CBD group where 1 humerus samples was not successfully excised. ROUT test was performed to detect outliers. 2-way ANOVA was performed followed by the Benjamini–Hochberg post-hoc test with FDR adjustment, q < 0.05.2-way ANOVA results are indicated under each graph. For posthoc test, different letters **(a–c)** indicate significant differences between groups.

Dual-energy x-ray absorptiometry was performed to evaluate BMC and aBMD after 18-weeks of CBD treatment. Compared to SS groups, CBD treatment partially reversed the OVX-induced decrease in whole-body aBMD (Figure 3A) and completely reversed the OVX-induced decrease in whole body BMC (Figure 3B). CBD did not alter whole-body BMC or aBMD of mice in the SS group (Figures 3A,B). Compared to the SS groups, VEH-treated OVX mice showed a significant decrease in femoral aBMD, which was rescued by CBD treatment (Figure 3C). Femoral BMC (Figure 3D), tibial aBMD and BMC (Figures 3E,F), humeral BMC (Figure 3H), and spinal BMC (Figure 3J) were not altered by OVX or CBD treatment. CBD did not affect OVX-induced reductions in aBMD of humerus (Figure 3G) and spine (Figure 3I).

**FIGURE 4:**
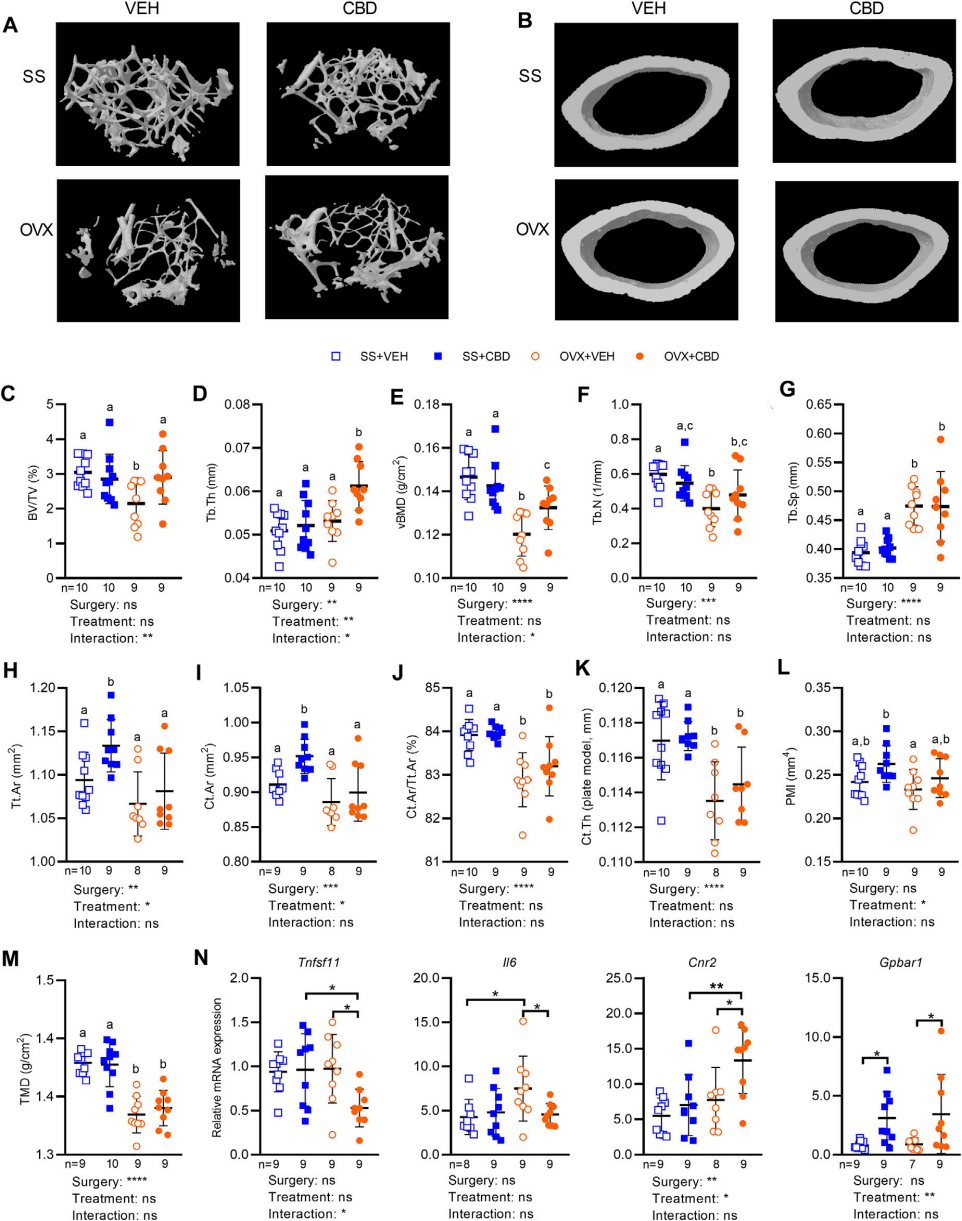
CBD improved femoral microstructure consistent with gene expression markers of reduced inflammation and less bone resorption. MicroCT images of femoral bone microstructure showing **(A)** trabecular bone and **(B)** cortical bone from SS and OVX groups after 18 weeks of VEH or CBD treatment. Femoral microstructure of trabecular bone showing **(C)** bone volume/tissue volume, BV/TV (%) **(D)** trabecular thickness, Tb.Th (mm) **(E)** volumetric bone mineral density, vBMD (g/cm^2^) **(F)** trabecular number, Tb.N (1/mm) **(G)** trabecular separation, Tb. Sp (mm) and cortical bone showing **(H)** mean total cross-sectional tissue area, Tt. Ar (mm^2^) **(I)** mean total cross-sectional bone area, Ct. Ar (mm^2^) **(J)** cortical bone fraction, Ct. Ar/Tt.Ar (%) **(K)** cortical thickness, Ct. Th (plate model, mm) **(L)** polar moment of inertia, PMI (mm^4^) **(M)** tissue mineral density, TMD (g/cm^2^). Data are presented as mean ± SD from 9 mice per OVX group and 10 mice per SS group. ROUT test was performed to detect outliers. 2-way ANOVA was performed (results are presented under graphs) followed by the Benjamini–Hochberg post-hoc test with FDR adjustment (q < 0.05) where different letters **(a–c)** indicate significant differences between groups. **(N)** qPCR analyses of femoral gene expression showing mRNA levels of RANKL, IL-6, CB2 and TGR5 was performed on 9 samples per group and the ROUT test was performed to detect outliers. Asterisk (*) indicates statistically differences determined by two-way ANOVA followed by Holm-Sidak post-hoc test to isolate differences due to surgery or treatment, *p < 0.05.

The resilience of bone to fracturing depends on bone microarchitecture, an indicator of bone strength and quality. Femur segments were therefore subject to microCT to assess the metaphysis area of trabecular (i.e., spongy) bone and the diaphysis area of cortical (i.e., compact) bone. Representative microCT images of femoral trabecular and cortical bone morphology for each treatment group are shown in Figures 4A,B. CBD treatment was able to reverse the OVX-induced reduction in trabecular bone volume fraction (BV/TV) or percentage of spongy bone tissue, raising it to levels comparable with SS groups (Figure 4C). CBD-treated OVX mice had higher trabecular thickness (Tb.Th) than VEH-treated OVX mice and SS groups (Figure 4D). Compared to SS groups, CBD partially reversed the OVX-induced decrease in volumetric bone mineral density (vBMD, Figure 4E). OVX groups had lower trabecular number (Tb.N) indicating lower density of trabeculae and higher trabecular separation (Tb.Sp) indicating more distance between trabeculae; however, these endpoints were not significantly changed by CBD (Figures 4F,G). Compared to the VEH-treated SS group, the mean total cross-sectional tissue area (Tt.Ar) and mean total cross-sectional bone area (Ct.Ar) of cortical bone were unaffected by OVX; however, the CBD-treated SS group showed an increase in these parameters (Figures 4H,I). CBD had no significant effect on the OVX-induced decrease in cortical bone fraction (Ct.Ar/Tt.Ar, Figure 4J) and cortical thickness (Ct.Th, Figure 4K). Polar moment of inertia (PMI), a measure of mechanical rigidity to torsion stress or twisting, was significantly lower in the VEH-treated OVX groups compared to the CBD-treated SS group, but otherwise similar between other groups (Figure 4L). CBD did not alter OVX-induced reduction in cortical tissue mineral density (TMD, Figure 4M).

Femoral gene expression was investigated to uncover possible molecular explanations for the improved bone phenotypes. We assessed tumor necrosis factor receptor superfamily (*Tnfrsf*) genes encoding members of the RANK/RANKL/OPG signaling pathway as well as tartrate-resistant acid phosphatase type 5 (TRAP), an osteoclastic marker of bone resorption (Udagawa et al., 2021). Compared to VEH-treated OVX mice, CBD-treated OVX mice showed a significant decrease in femoral gene expression of *Tnfrsf11*, encoding RANKL (Figure 4N), indicating a disruption of the RANKL-RANK interaction and decreased bone resorption. Expression of *Tnfrsf11a, Tnfrsf11b*, and *Acp5* encoding RANK, OPG, and TRAP respectively, was not significantly altered by ovariectomy or CBD (Supplementary Figure S3B). Compared to the VEH-treated OVX group or the CBD-treated SS group, the OPG/RANKL mRNA ratio was significantly higher in CBD-treated OVX mice (Supplementary Figure S3C) suggesting bone formation and inhibition of osteoclast differentiation (Xu et al., 2019). The RANKL/OPG mRNA ratio was not different between the four groups (Supplementary Figure S3C) suggesting similar bone resorption and turnover (Jura-Półtorak et al., 2021). Compared to the VEH-treated SS group, femoral expression of *Il6* was increased in VEH-treated OVX mice, and this effect was reversed by CBD-treatment (Figure 4N). *Il1b* and *Tnf* levels were similar across groups (Supplementary Figure S3B).

Stimulation of endocannabinoid receptor CB2 was reported to inhibit RANKL release leading to reduced osteoclastogenesis while stimulation of CB1 and TRPV1 activates osteoclastogenesis and bone resorption (Idris et al., 2005; Rossi et al., 2015). CBD is an antagonist of GPR55 that has been shown to inhibit bone resorption in male mice (dosed at 10 mg/kg, 3 times per week), although changes were not significant (Whyte et al., 2009). CBD-treated OVX mice had increased femoral mRNA levels of *Cnr2*, encoding CB2 (Figure 4N), while mRNA levels of *Cnr1* (encoding CB1), Trpv1, and Gpr55 were similar among the four treatment groups (Supplementary Figure S3B).

BA receptors TGR5 and FXR are expressed in osteoblasts and osteoclasts where they regulate bone metabolism (Zhao et al., 2020). Activation of FXR has been shown to suppress osteoclast differentiation, increase osteoblast differentiation (Cho et al., 2013), and promote expression of TGR5 (Pathak et al., 2017). TGR5 inhibited osteoclast differentiation while loss of TGR5 promoted osteoclast differentiation and bone resorption (Li et al., 2019). Compared to VEH-treatment, CBD-treated OVX and SS groups had increased levels of *Gpbar1* mRNA encoding TGR5 (Figure 4N) suggesting suppression of osteoclast differentiation. CBD did not alter expression of *Nr1h4* or the FXR target gene, *Nr0b2* encoding small heterodimer protein (SHP) (Supplementary Figure S3B).

### 3.4 Cannabidiol Did Not Alter Short Chain Fatty Acids But Induced Bile Acid Changes Consistent With Decreased Inflammation and Improved Glucose and Bone Metabolism

SCFAs levels were not altered by ovariectomy or CBD treatment (Supplementary Figure S4). Bile acid (BA) species have been reported to alter gut bacteria (Ridlon et al., 2014), regulate inflammation (Chen et al., 2019), glucose metabolism (Ahmad and Haeusler, 2019), and bone turnover (Cho et al., 2013). Primary and secondary BAs (i.e., PBAs and SBAs) in ileum content and serum were therefore quantified by LC-MS using external and internal standards.

Serum levels of total BAs, PBAs, SBAs, conjugated BAs, and unconjugated BAs were similar in VEH- and CBD-treated OVX groups (Table 1). Compared to the VEH-treated SS group, total BAs and PBAs were significantly decreased in serum of the CBD-treated SS group (Table 1); specifically individual PBAs that were decreased in the serum of the CBD-treated SS group included tauroursodeoxycholic acid (TUDCA), tauro-α-muricholic acid (TαMCA) and ursodeoxycholic acid (UDCA). Compared to VEH-treated OVX mice, TUDCA showed a trending increase in serum of CBD-treated OVX mice (q = 0.11; Table 1). TUDCA is FDA-approved for treatment of cholestatic liver disease and has also been reported to promote differentiation of osteoblasts, improve markers of bone quality, suppress inflammatory cytokines, and improve glucose tolerance (Yanguas-Casás et al., 2017; Kim et al., 2018; Zangerolamo et al., 2021). Glycocholic acid (GCA), a PBA which has been identified as a biomarker for hepatocellular carcinoma (Li H. et al., 2017) was decreased to non-detectable levels in the serum of CBD-treated OVX mice compared to VEH-treated OVX mice (Table 1). Serum TαMCA and TωMCA, which are FXR antagonists associated with prevention of hepatic cholestasis (Takikawa et al., 1997; Sayin et al., 2013), were decreased in CBD-treated OVX mice, compared to VEH-treated OVX mice (Table 1). Compared to the VEH-treated SS mice, DCA was reduced in the CBD-treated SS group. High deoxycholic acid (DCA) levels have been associated with low BMD in people over 60 (Jovanovich et al., 2018), with colorectal cancer (Bayerdörffer et al., 1995), and with gut dysbiosis, intestinal inflammation, increased bile acid pool, and lower intestinal FXR activity (Wang et al., 2020; Xu et al., 2021a; Xu et al., 2021b).

In ileal content, the concentrations of total BAs, PBAs, and SBAs were similar among treatment groups. SBAs in the CBD-treated OVX group trended lower than the VEH-treated OVX group (q = 0.06, *p* = 0.02), perhaps due to reduced hyocholic acid (HCA) and ωMCA in CBD-treated OVX mice (Table 1). Compared to SS groups, the VEH-treated OVX group had increased concentration of glycine-conjugated BAs; however, levels were reduced to that of the SS groups in the CBD-treated OVX group (Table 1). Comparison of individual ileal content bile acids in VEH-treated SS and OVX groups revealed that glycoursodeoxycholic acid (GUDCA), glycochenodeoxycholic acid (GCDCA), GCA, HCA, ωMCA, and taurohyodeoxycholic acid (THDCA) were increased in OVX mice; however, in CBD-treated OVX mice the concentrations of these BAs were decreased to levels more consistent with SS groups (Table 1). Increased glycine-conjugated BA in small intestine, specifically GCDCA and GCA, has been correlated with increased intestinal inflammation in indomethacin-treated rats (Lázár et al., 2021). Taurocholic acid (TCA) trended higher in VEH-treated OVX mice compared to the SS groups (q = 0.09) but was significantly lower in the CBD-treated OVX mice (Table 1). Within the SS groups, CBD did not significantly change the profile of ileal BAs except for decreased taurolithocholic acid (TLCA, Table 1).

### 3.5 Cannabidiol-Treated Ovariectomized Mice Develop Intestinal Bloom in *Lactobacillus* Species

**FIGURE 5:**
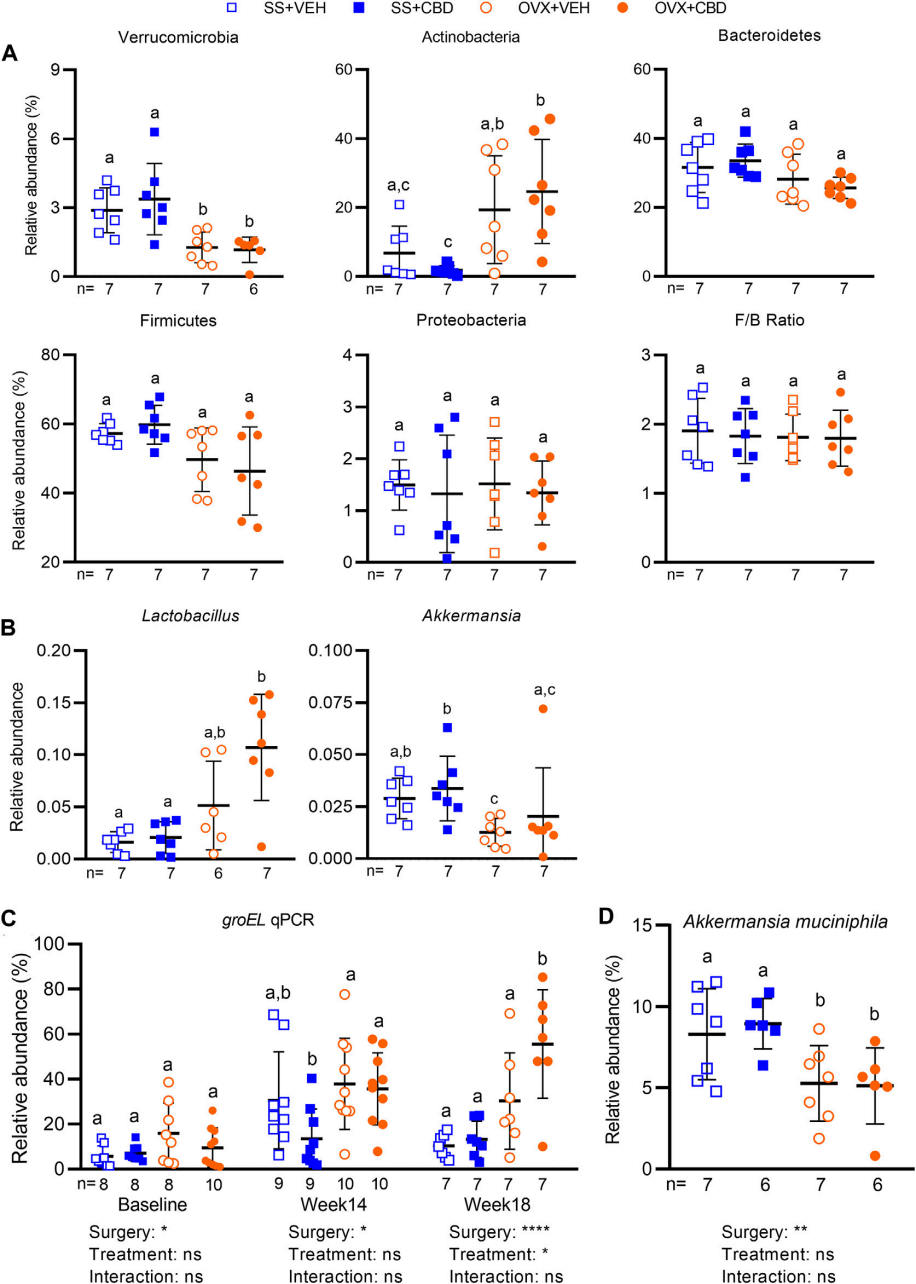
CBD altered the gut microbiota and increased relative abundance of *Lactobacillus* sp. Relative abundance of amplicon sequence variants (ASVs) identified in fecal gut microbiota samples collected after 18 weeks of VEH or CBD treatment (*n* = 7 mice/group) classified at the **(A)** phyla and **(B)** genera level using a Naïve-Bayes trained taxonomic classifier from the Silva database. ROUT test was performed to detect outliers. Different letters **(a–c)** indicate significant difference as determined by the non-parametric Kruskal–Wallis test for non-normally distributed data followed by Benjamini–Hochberg post-hoc test with FDR-adjustment, q < 0.05. **(C)** qPCR using primers specific to variable region of *groEL* gene to confirm relative abundance of *Lactobacillus* spp. in fecal samples collected at baseline (*n* = 8–10 mice/group), week 14 (*n* = 9–10 mice/group), and week 18 (*n* = 7 mice/group) after VEH or CBD treatment. **(D)** qPCR to assess relative abundance of *Akkermansia muciniphila* at week 18 (*n* = 7 mice/group) using *A. muciniphila* specific primers. Data in C and D were normally distributed. ROUT test was performed to detect outliers. Different letters **(a,b)** indicate significant difference determined by two-way ANOVA followed by Benjamini–Hochberg post-hoc test with FDR-adjustment, q < 0.05.

16S rRNA amplicon sequencing was performed to assess fecal microbial communities after 18 weeks of CBD treatment. Faith’s phylogenetic diversity index showed that all groups had fewer amplicon sequence variants (ASVs) relative to the VEH-treated SS group (Supplementary Figure S5A). Richness of the CBD-treated OVX group was lower than the VEH-treated SS group but otherwise richness, Shannon index, and Pielou’s evenness metrics were similar among the four groups (Supplementary Figure S5A). With respect to *β*-diversity, Jaccard and Bray Curtis dissimilarity indices and weighted unifrac showed that SS and OVX groups were significantly separated along principal coordinate 1 (PC1) but communities were not distinguishable based on CBD treatment (Supplementary Figure S5B). Unweighted unifrac, accounting for presence/absence of taxa only, showed the VEH-treated SS group separated from the other three treatment groups along PC1 (Supplementary Figure S5B). Compared to SS groups, OVX mice had decreased relative abundance of phylum Verrucomicrobia and a trend of increased Actinobacteria, but Proteobacteria, Bacteroidetes, Firmicutes, and the Firmicutes/Bacteroidetes ratio were similar among all groups (Figure 5A).

At the genera level, compared to the VEH-treated OVX group, the CBD-treated OVX mice had a trending increase in the relative abundance of *Lactobacillus* (Figure 5B). Supplementation of *Lactobacillus* probiotics was shown to reduce bone loss and inflammation in OVX rodents (Rizzoli and Biver, 2020; Yang et al., 2020; Sapra et al., 2021) and in postmenopausal women (Nilsson et al., 2018; Jansson et al., 2019). To confirm the increase in fecal *Lactobacillus* species, qPCR was performed using primers specific to the *groEL* gene, a single copy gene encoding heat stress proteins, which was reported to identify *Lactobacillus* in foods and fecal samples with higher resolution than the 16S rRNA gene (Xie et al., 2019). Compared to VEH-treated OVX mice, CBD-treated OVX mice had higher relative abundance of *groEL* sequences confirming that *Lactobacillus* was increased after 18 weeks of treatment (Figure 5C).

Compared to SS groups, *Akkermansia* was decreased in OVX groups and qPCR confirmed that there was lower relative abundance of *Akkermansia muciniphila* (Figures 5B,D). *A. muciniphila* has been correlated with metabolic health therefore its reduction in OVX mice may contribute to the increased intestinal inflammation and reduced glucose tolerance in E2 deficiency (Cani and de Vos, 2017). Compared to the CBD-treated SS group, the CBD-treated OVX group also showed increased abundance of *Bifidobacterium* and Coriobacteriaceae UCG-002, but decreased relative abundances of *Enterococcus*, *Romboutsia*, Peptococcaceae *Clostridium sensu strico* 1 and *Enterohabdus* (Supplementary Figure S5C). The CBD-treated SS group had higher relative abundance of *Enterococcus* compared to all other groups and VEH-treated OVX mice had lower relative abundance of *Dubosiella* compared to the SS groups (Supplementary Figure S5C).

## 4 Discussion

In this study we showed that CBD treatment can protect against OVX-induced inflammation, bone loss, impaired energy metabolism, and glucose intolerance. These CBD-induced phenotypic improvements occurred in association with beneficial alterations in the gut microbiota and BA profiles. Other studies in OVX mice have shown that E2 deficiency promotes intestinal inflammation (Li et al., 2016; Collins et al., 2017; Yu M. et al., 2021). In our study VEH-treated OVX mice did not show increased gene expression of intestinal inflammatory markers (Figure 2, IL6, TNF or IL1β) compared to the VEH-treated SS group. Nonetheless, consistent with its reported anti-inflammatory effects (Rajan et al., 2016; Couch et al., 2017); CBD treatment diminished the expression of proinflammatory markers in the intestinal tissues of both SS and OVX mice.

CBD treatment improved OGT (Figure 1B) and EE (i.e., heat production, Figure 1F) in OVX mice without increasing physical activity (Supplementary 2), or altering intestinal markers related to glucose metabolism (Supplementary Figure S3A). It is possible that CBD increased EE by promoting the metabolic activity of brown adipose tissue (BAT) or by promoting the conversion of white adipocytes into beige or brown adipocytes, where color indicates the abundance of iron-rich mitochondria (Jung et al., 2019). BAT oxidizes fatty acids to produce heat, a process termed non-shivering thermogenesis (NST). BAT activity can be stimulated by cold weather (i.e., cold-induced thermogenesis), food consumption (diet-induced thermogenesis), or non-caloric phytochemicals such as capsaicin, caffeine, and catechins (Saito et al., 2020). Specific members of the gut microbiota, including *Lactobacillus* sp., have also been associated with BAT activation (Moreno-Navarrete and Fernandez-Real, 2019; Yoon et al., 2020; Kang et al., 2022). *In vitro* CBD treatment was shown to induce browning of cultured 3T3-L1 adipocytes by increasing their expression of BAT-specific marker genes (Parray and Yun, 2016). Brown adipocytes also take up glucose from circulation for *de novo* synthesis of free fatty acids to fuel NST (Wang et al., 2021) therefore a CBD-induced increase in brown adipocyte activity or quantity may also provide explanation for the observed improvement in OGT (Figure 1B). Our future work will explore these potential CBD-induced mechanisms.

Germ-free mice are protected from ovariectomy-induced bone loss compared to conventional or colonized mice emphasizing that the gut microbiota is a regulator of bone mass (Li et al., 2016). It was recently shown in OVX mice that gut-derived TNF-producing T cells (TNF + T cells) migrate to bone marrow and then chemoattract gut-derived IL17-producing T cells (Th17 cells) to bone where they both promote RANKL activity and bone loss (Yu M. et al., 2021). CBD treatment of OVX mice reduced femoral gene expression of RANKL and IL6 consistent with reduced bone loss (Figure 4N), possibly due to less Th17 cell activation. The present study did not separate bone marrow from cortical bone therefore the lower inflammation could be due to multiple cell types. Future work should investigate the response of individual bone and immune cell types.

Interestingly, CBD promoted a bloom in *Lactobacillus* species. Prior studies indicated that probiotic *Lactobacillus* treatment may protect against bone loss by reducing gut permeability and levels of proinflammatory cytokines in the gut, circulation, and bone (Nilsson et al., 2018; Jansson et al., 2019; Rizzoli and Biver, 2020; Yang et al., 2020; Sapra et al., 2021). A recent meta-analysis assessed the effects of probiotic supplementation on BMD and bone turnover markers for postmenopausal women and suggested that probiotics may increase lumbar BMD (Yu J. et al., 2021). E2 deficiency is associated with decreased relative abundance of *A. muciniphila*, a microbe that has been correlated with improving metabolic phenotypes such as insulin resistance, glucose tolerance, and dyslipidemia (Brahe et al., 2015). Pasteurized *A. muciniphila* (pAkk) has shown benefit for obesity and insulin resistance in mice and humans, at least partially due to the activity of outer membrane protein Amuc_1,100 (Plovier et al., 2017; Depommier et al., 2019). It was recently shown that although pAkk treatment reduced fat mass gain in SS and OVX mice, pAkk reduced bone mass in ovary-intact mice and did not protect OVX mice from bone loss (Lawenius et al., 2020). Other studies have reported an increased Firmicutes/Bacteroidetes ratio in OVX mice (Wen et al., 2020); however, we did not observe this in our study, possibly due to differences in mouse strains, vendors, and/or gut microbial community. Compared to SS groups, OVX mice had an increase in relative abundance of Verrucomicrobia, as previously reported (Wen et al., 2020). Knowledge of gut bacterial changes and functions at the strain level will be needed for better understanding of bacteria-host relationships.

CA and CDCA are the main PBAs produced in humans and rodents. In mice, but not humans, CDCA undergoes 6β-hydroxylation to produce muricholic acids (i.e., αMCA, βMCA). To increase solubility, PBAs undergo N-acyl amidation with amino acids to produce predominantly taurine conjugates in mice and mainly glycine conjugates in humans (Jäntti et al., 2014; Winston and Theriot, 2020). Once secreted in the intestine, gut bacteria transform PBA into SBA further expanding the repertoire of BA species that can modulate host BA receptors throughout the body. In the process of enterohepatic circulation, over 95% of BAs are reabsorbed in the intestine, recirculated to the liver via the hepatic portal vein, and recycled into bile. About 5% of BAs are excreted but a small fraction of BAs enters systemic circulation, which allows BA signaling to take place in organs and tissues beyond the gut-liver axis, such as bone (Winston and Theriot, 2020). Bacteria produce SBAs via deconjugation (i.e., deamidation), dehydroxylation, dehydrogenation, and epimerization reactions (Wahlström et al., 2016). Actinobacteria and *Lactobacillus* sp. harbor BSH genes (O'Flaherty et al., 2018; Jones et al., 2008) that deconjugate BAs to produce SBAs, which have weaker detergent properties creating a less toxic environment for gut bacteria. Unconjugated BAs are less efficiently reabsorbed than conjugated BAs and therefore will pass to the colon where they can be further metabolized to SBAs and interact with TGR5 in colonocytes (Cipriani et al., 2011; Sorrentino et al., 2020; Dong et al., 2021). In both CBD-treated SS and OVX groups the levels of unconjugated BAs were elevated in ileal content, albeit non-significantly, suggesting that increased amounts can enter the colon for conversion to SBAs. The interaction of SBA and TGR5 in the colon has been shown to promote expression of tight junction proteins, improve gut barrier integrity, and increase GLP-1 secretion resulting in improved glucose tolerance (Harach et al., 2012; Kim and Fang, 2018). Compared to VEH-treated SS mice, expression of tight junction proteins was lower in VEH-treated OVX mice; however, CBD-treated OVX mice had increased expression of colonic *Tjp1*, trending increases in ileal *Tjp1* and *Ocln* (Figure 2), and improved glucose clearance (Figure 1).

Compared to the VEH-treated SS group, the VEH-treated OVX group showed high levels of several BAs in ileal content (i.e., GUDCA, GCDCA, GCA, HCA, ωMCA, THDCA); however, these same BAs were not higher in serum. It is unclear whether these elevated BAs are reabsorbed into enterohepatic circulation or excreted and how they may contribute to the negative phenotypes in OVX mice. CBD-treated OVX mice had increased femoral expression of *Gpbar1* (TGR5), which is known to promote bone mass (Li et al., 2019). Whole body deletion of TGR5 in ovary-intact mice aged 7 months or 4-month-old OVX mice resulted in decreased bone mass compared to wild type controls indicating that TGR5 plays a positive role in promoting bone mass in aged or OVX mice with osteoporosis (Li et al., 2019). Loss of TGR5 promoted osteoclast differentiation and bone resorption (Li et al., 2019). We do not yet have direct evidence for which BAs may activate femoral TGR5 in the CBD-treated mice; however, circulating TUDCA was increased in CBD-treated OVX mice and may play a role. Oral administration of TUDCA to OVX mice resulted in distal femurs with preserved trabecular microstructure and *in vitro* TUDCA treatment increased osteoblasts viability and differentiation (Ahn et al., 2020). *In vitro*, TUDCA inhibited LPS-induced inflammation in RAW 264.7 macrophages, BV2 microglial cells, and bone marrow-derived macrophages (BMMs) and *in vivo* promoted recovery and suppressed inflammatory cytokines in rats with spinal cord injury (Kim et al., 2018). TUDCA activated TGR5 and had an anti-inflammatory effect on microglial cells (Yanguas-Casás et al., 2014; Yanguas‐Casás et al., 2017). TUDCA improved glucose tolerance and insulin sensitivity in streptozotocin-induced model of Alzheimer’s disease (Zangerolamo et al., 2021).

Endocannabinoid receptors type-1 (CB1) is expressed mainly in the central nervous system while CB2 is expressed mainly in immune cells and peripheral tissues (Bab and Zimmer, 2008). CB2 is expressed in osteoblasts, osteoclasts, and osteocytes (Li et al., 2022). CBD attenuated inflammatory cytokines and promoted osteogenic differentiation of LPS-treated bone marrow mesenchymal stem cells via CB2 and p38 mitogen-activated protein kinase (MAPK) signaling pathways (Li et al., 2022). Ofek et al. reported lower bone mass in CB2 knock-out mice compared to WT C57BL/6J littermates (Ofek et al., 2006). Furthermore, they reported that the CB2 agonist HU308, a CBD derivative compound, could attenuate ovariectomy-induced bone loss in mice and promote osteoblastogenesis and inhibit osteoclastogenesis *in vitro* (Ofek et al., 2006). In contrast, Idris et al. reported that WT (C57BL/6) and CB2 knockout littermates showed no difference in peak bone mass, but OVX CB2 knockout mice were protected from bone loss compared to OVX WT mice (Idris et al., 2008). In addition, Idris et al. showed that CB2-selective agonists, JWH133 and HU308, could stimulate osteoclast formation (Idris et al., 2008). The reasons for the conflicting results reported for CB2 regulation of bone mass are unclear.

CBD efficacy has been evaluated in several rodent disease models and a common observation is decreased levels of inflammatory markers in circulation and different tissue types (Nichols and Kaplan, 2020). Most studies have delivered CBD via intraperitoneal injection (IP) (Nichols and Kaplan, 2020). For example, CBD reduced intestinal inflammation in a murine model of colitis where mice were injected intraperitoneally (IP) with 5 mg CBD/kg (Borrelli et al., 2009) and in an LPS-induced murine model of intestinal motility disturbance where mice were IP injected with 5 mg CBD/kg (Lin et al., 2011). We used a higher dose (25 mg/kg) due to the lower CBD bioavailability expected with oral administration (Millar et al., 2018; Perucca and Bialer, 2020) with no observed negative effects. This murine dose would translate to a human equivalent dose of 2.03 mg/kg body weight or 120 mg/day for a 60 kg adult female (Nair and Jacob, 2016). Studies have reported safe use of much higher daily doses of oral CBD (Larsen and Shahinas, 2020).

In conclusion, our results indicate that CBD treatment of OVX mice impacts the immune system and the gut microbiota to improve energy metabolism and bone homeostasis. These data indicate that CBD modulates a gut-bone axis to favorably alleviate several chronic disease symptoms of postmenopause.

## Data Availability

The datasets presented in this study can be found in online repositories. The names of the repository/repositories and accession number(s) can be found below: https://ddbj.nig.ac.jp/search, PRJDB13331.
