# Corrigendum: Cannabidiol-treated ovariectomized mice show improved glucose, energy, and bone metabolism with a bloom in *Lactobacillus*

**DOI:** 10.3389/fphar.2022.1026500

**Published:** 2022-10-11

**Authors:** Ke Sui, Kevin M. Tveter, Fiona G. Bawagan, Patricia Buckendahl, Savannah A. Martinez, Zehra H. Jaffri, Avery T. MacDonell, Yue Wu, Rocio M. Duran, Sue A. Shapses, Diana E. Roopchand

**Affiliations:** ^1^ Department of Food Science, NJ Institute for Food Nutrition and Health (Rutgers Center for Lipid Research and Center for Nutrition Microbiome and Health), Rutgers, The State University of New Jersey, New Brunswick, NJ, United States; ^2^ Molecular Imaging Center, Rutgers, The State University of New Jersey, New Brunswick, NJ, United States; ^3^ Department of Nutritional Sciences, NJ Institute for Food Nutrition and Health, Rutgers, The State University of New Jersey, and the Department of Medicine, Rutgers-RWJ Medical School, New Brunswick, NJ, United States

**Keywords:** cannabidiol, estrogen deficiency, inflammation, osteoporosis, gut microbiota, bile acids

In the published article, there was an error in **affiliation 3**. Instead of “Department of Nutritional Sciences, NJ Institute for Food Nutrition and Health (Center for Human Health and Performance), Rutgers, The State University of New Jersey, New Brunswick, NJ, United States”, it should be “Department of Nutritional Sciences, NJ Institute for Food Nutrition and Health, Rutgers, The State University of New Jersey, and the Department of Medicine, Rutgers-RWJ Medical School, New Brunswick, NJ, United States”.

The authors apologize for this error and state that this does not change the scientific conclusions of the article in any way. The original article has been updated.

