# Supplementary material for: Cannabidiol-Treated Ovariectomized Mice Show Improved Glucose, Energy, and Bone Metabolism With a Bloom in Lactobacillus
Source: Front Pharmacol. 2022 Jun 21;13:900667. doi: 10.3389/fphar.2022.900667 (PMC9255917; doi:10.3389/fphar.2022.900667)
Supplement: Supplementary file 1 [file DataSheet1.docx]

**Supplementary Figure 1**


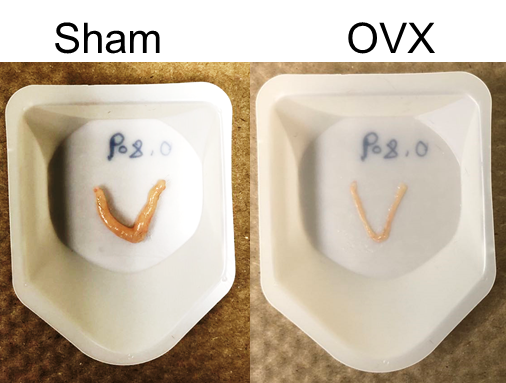

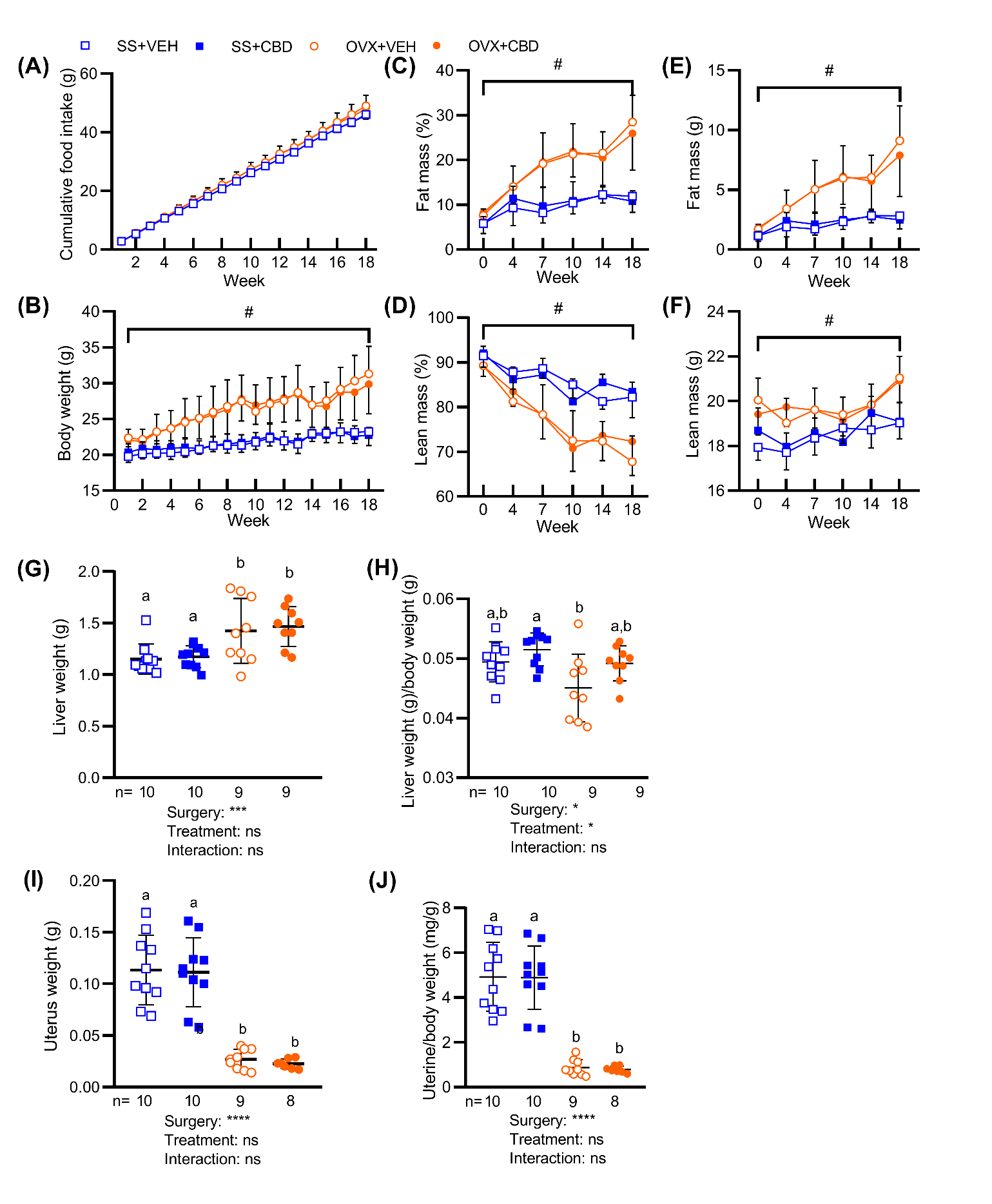


**Supplementary Figure 2**

#
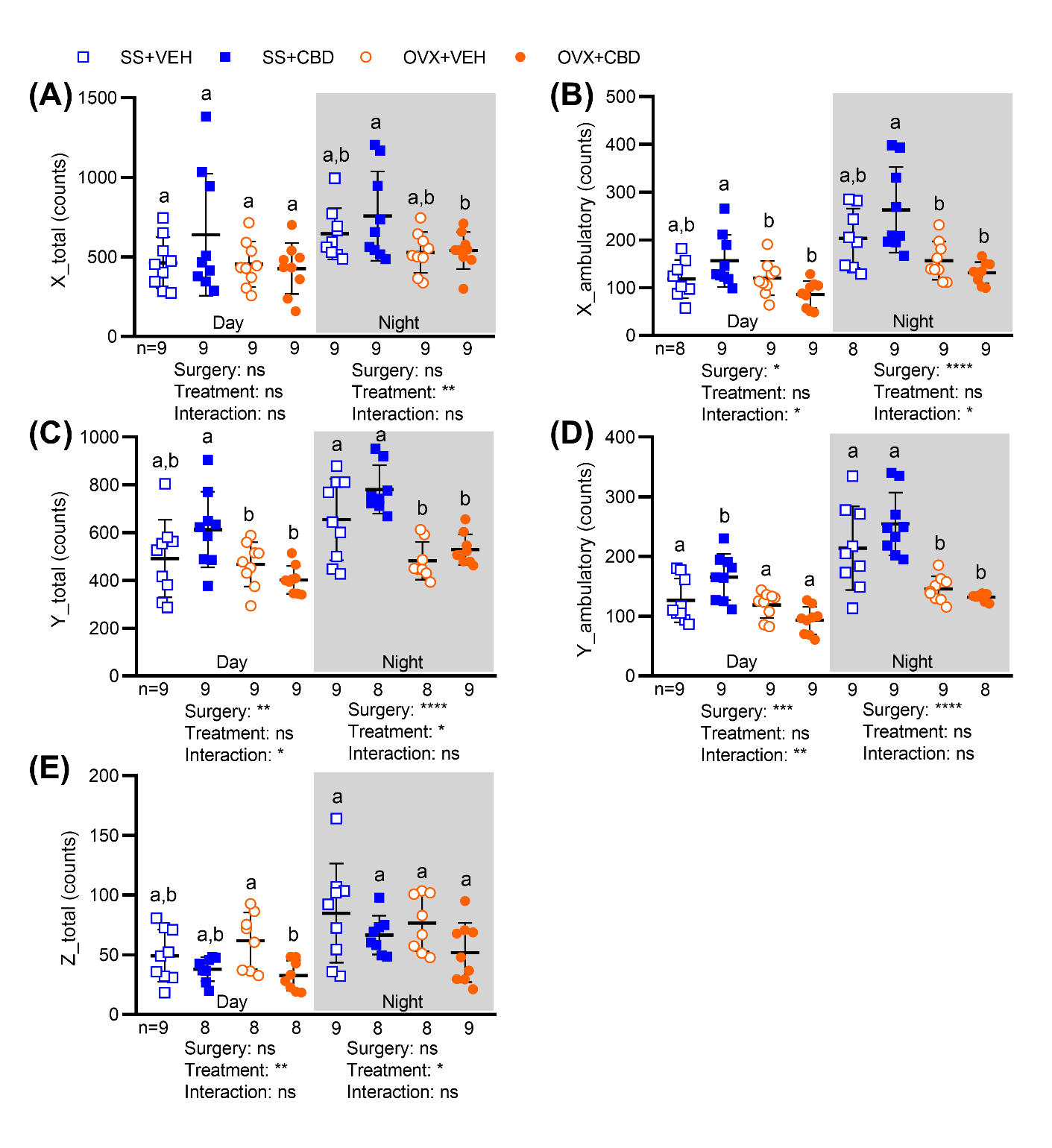


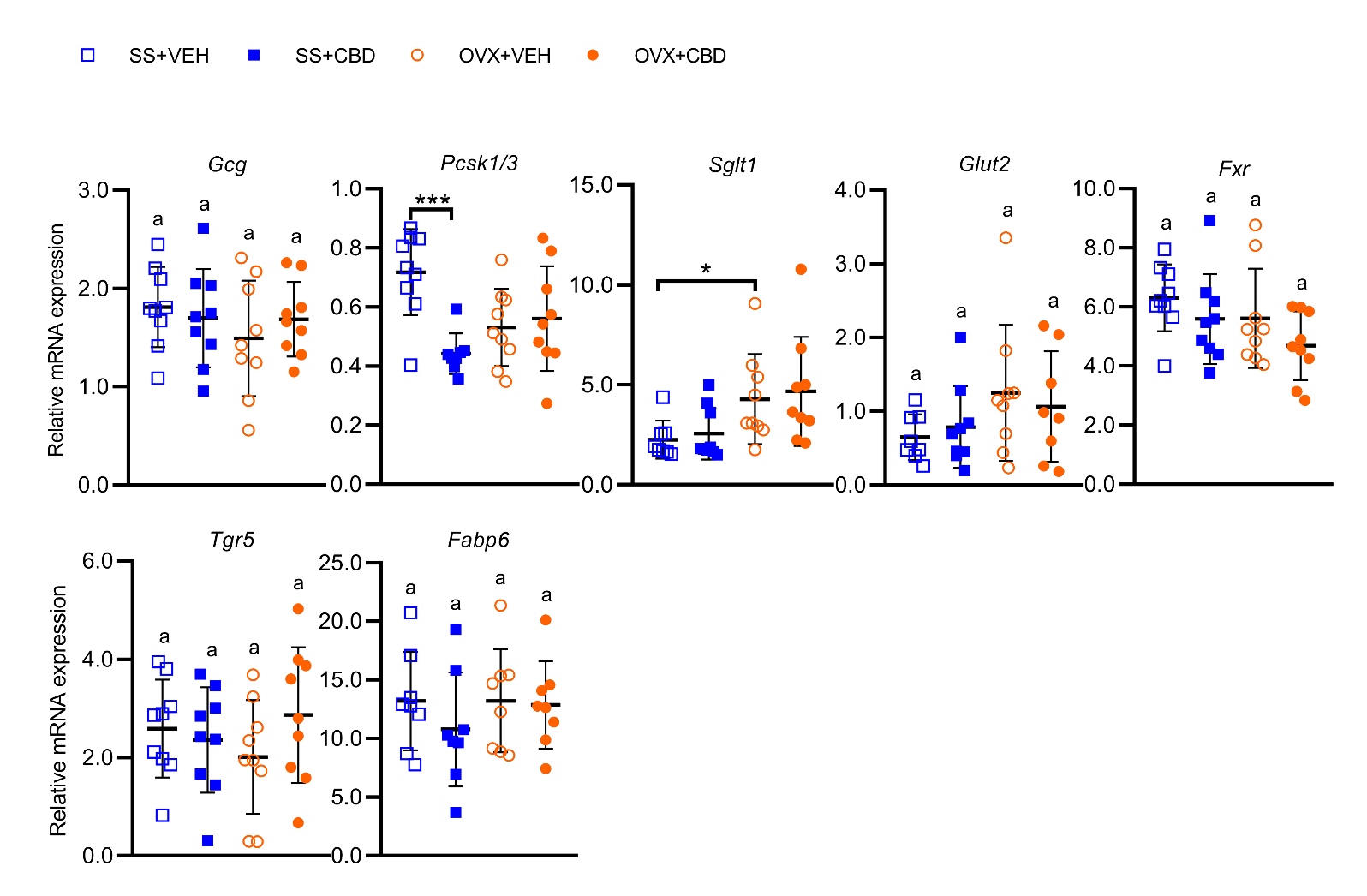
**Supplementary Figure 3**

**(A) Ileum**

**
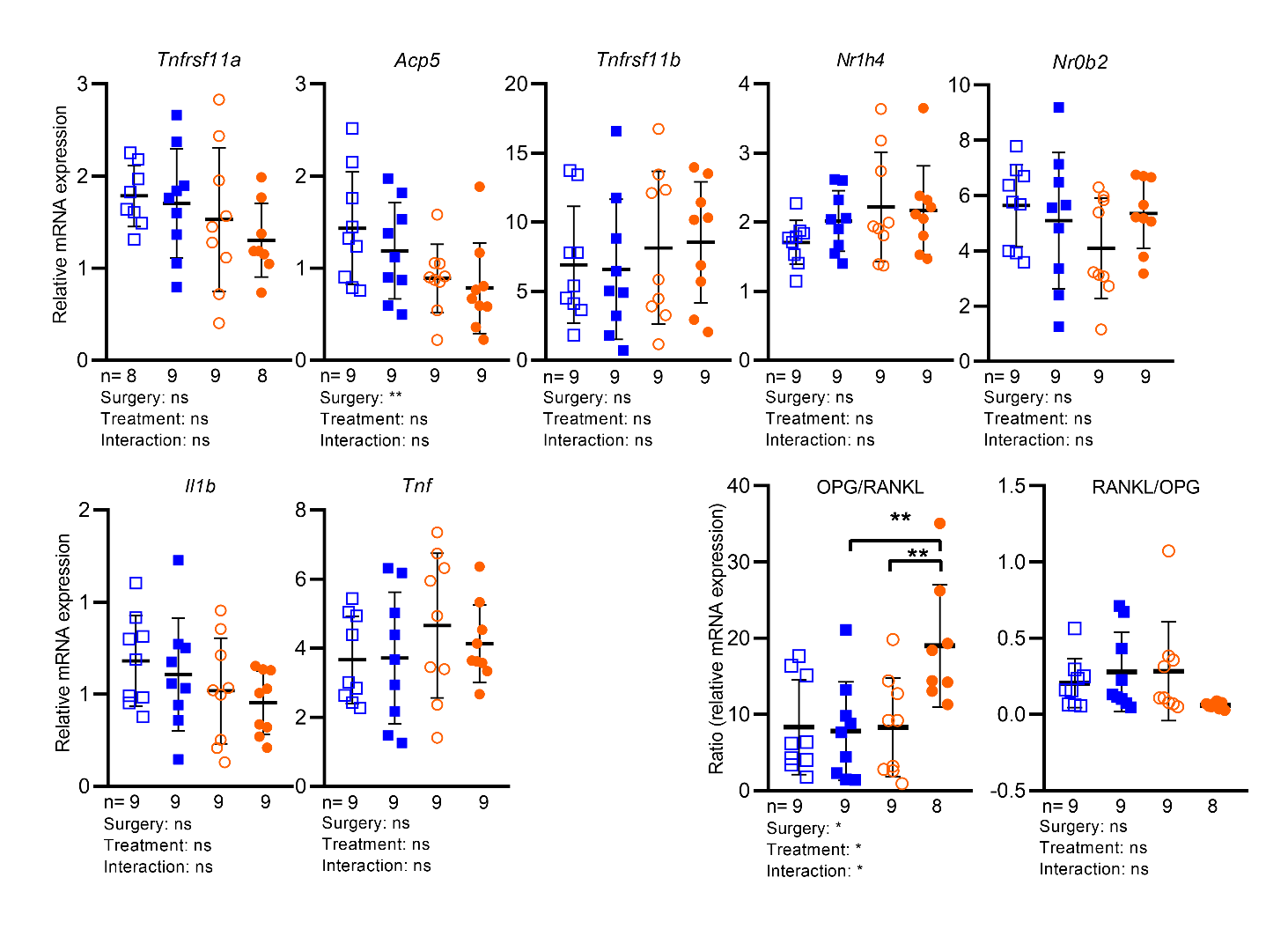

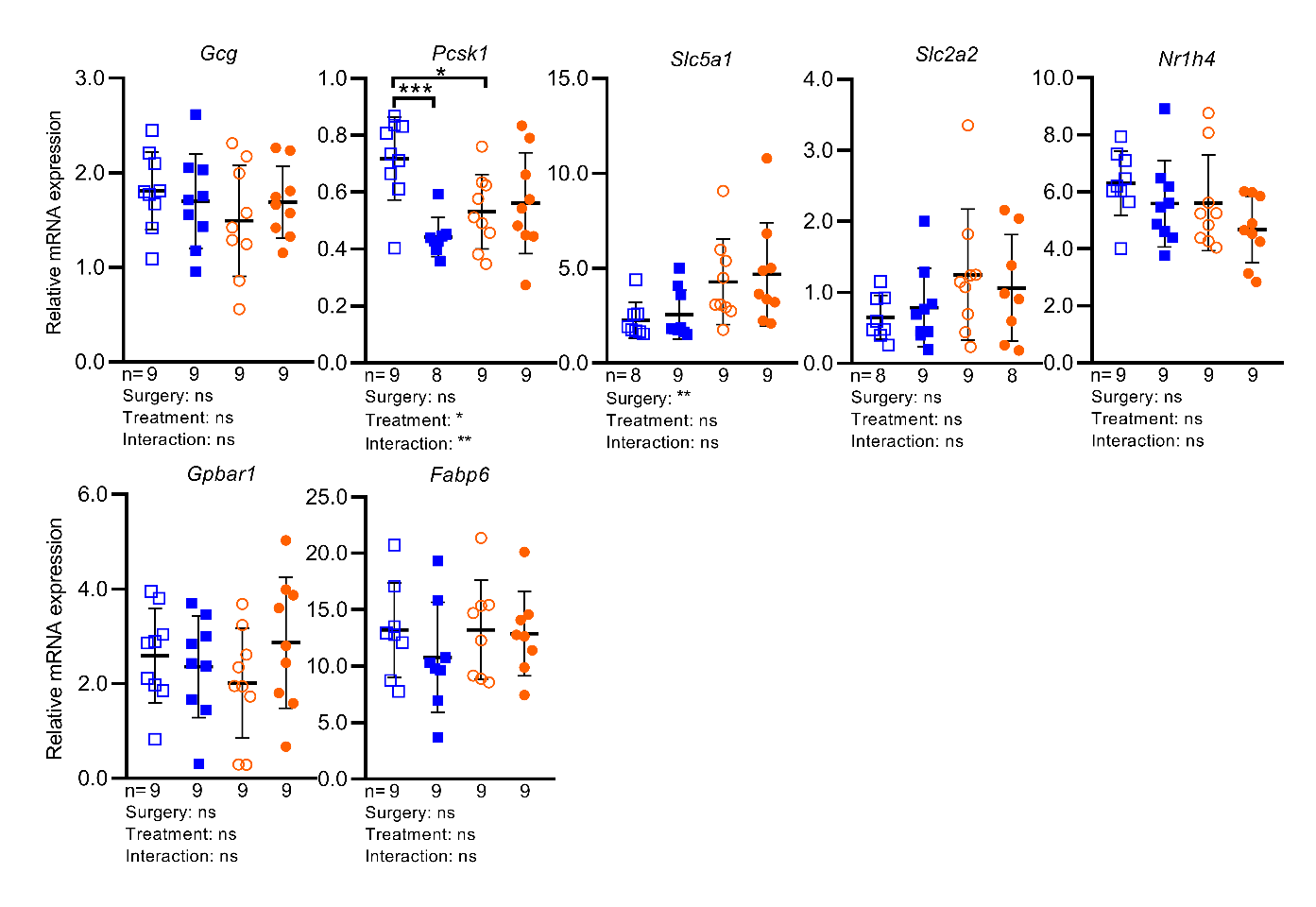
**

**(C)**

**(B) Femur**

**Supplementary Figure 4**

**
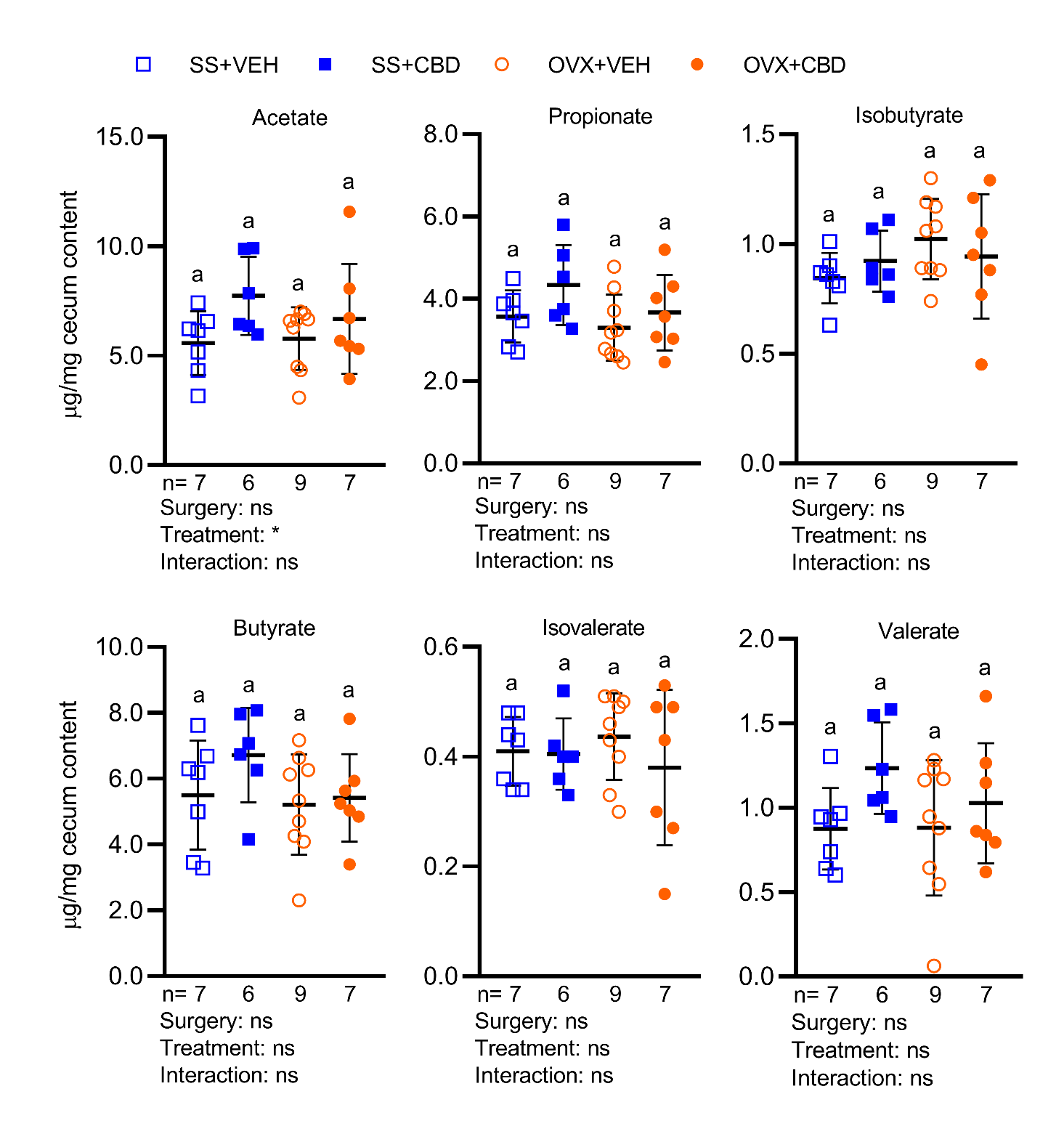
**

**Supplementary Figure 5**

**(A)**

**
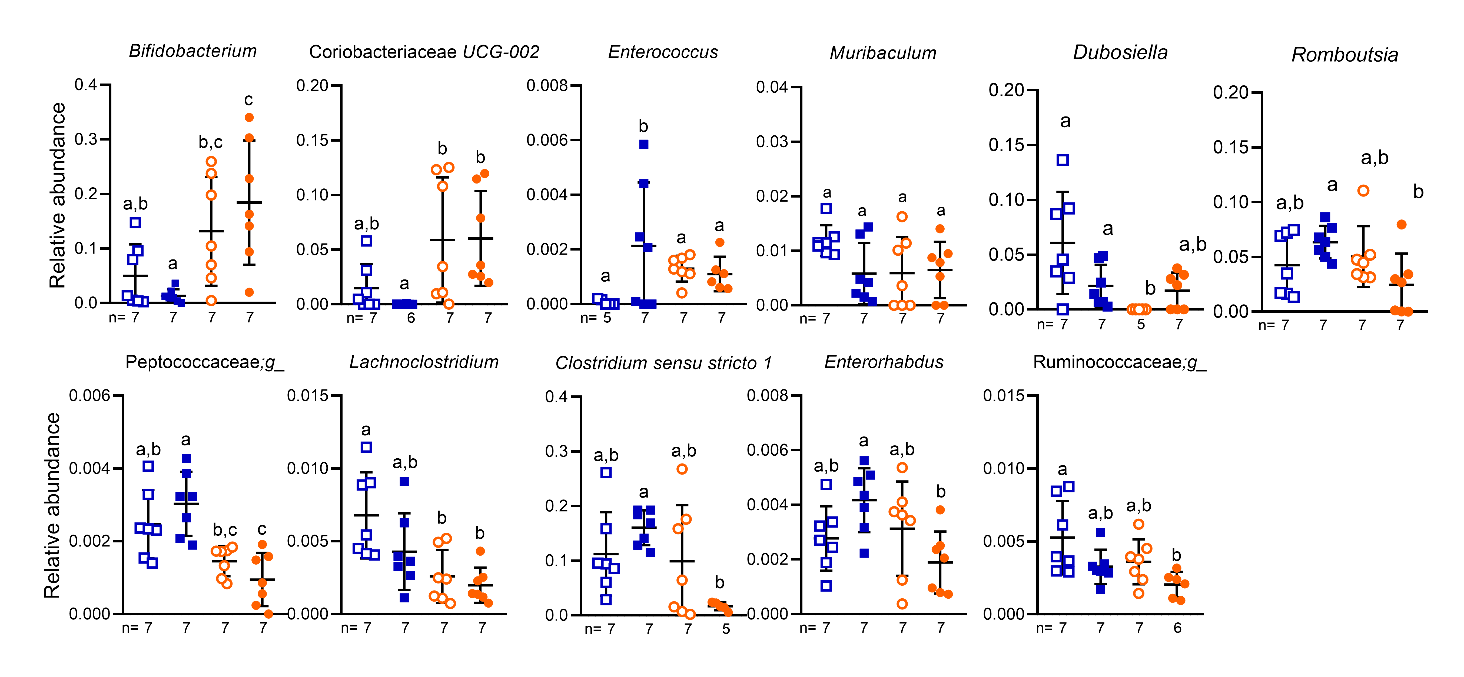

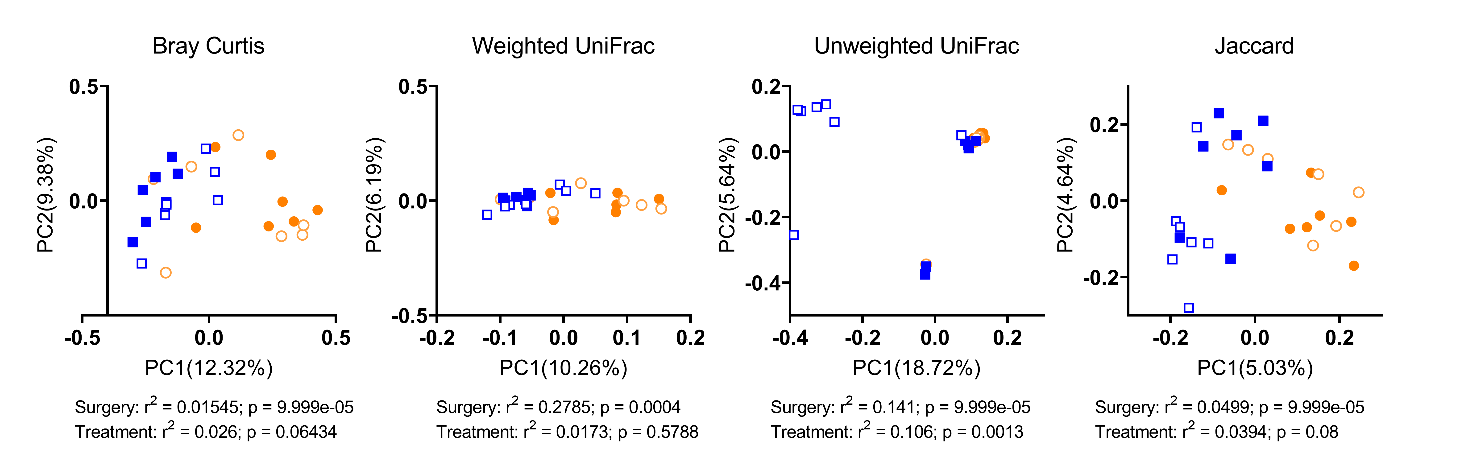

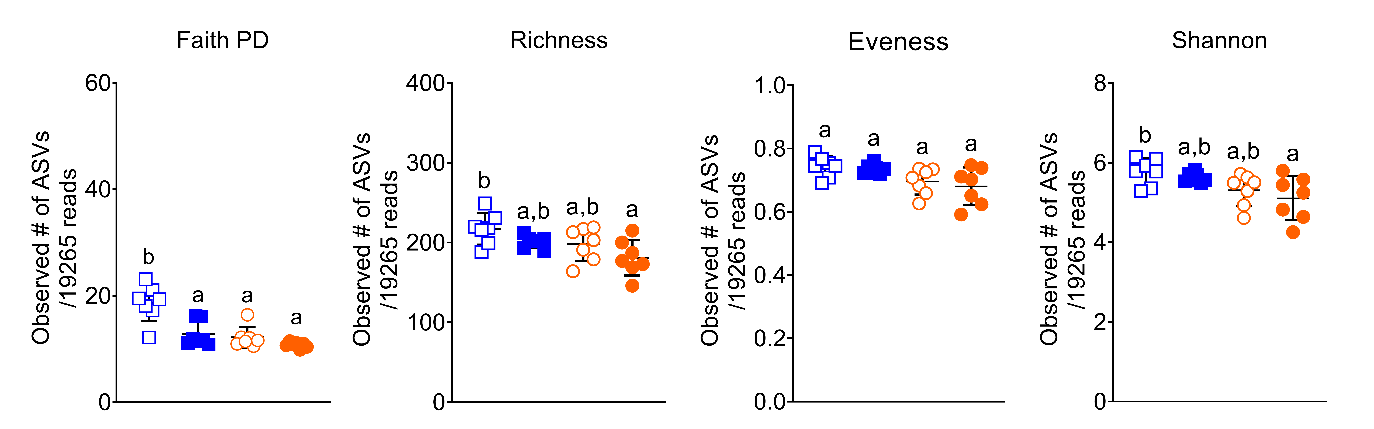

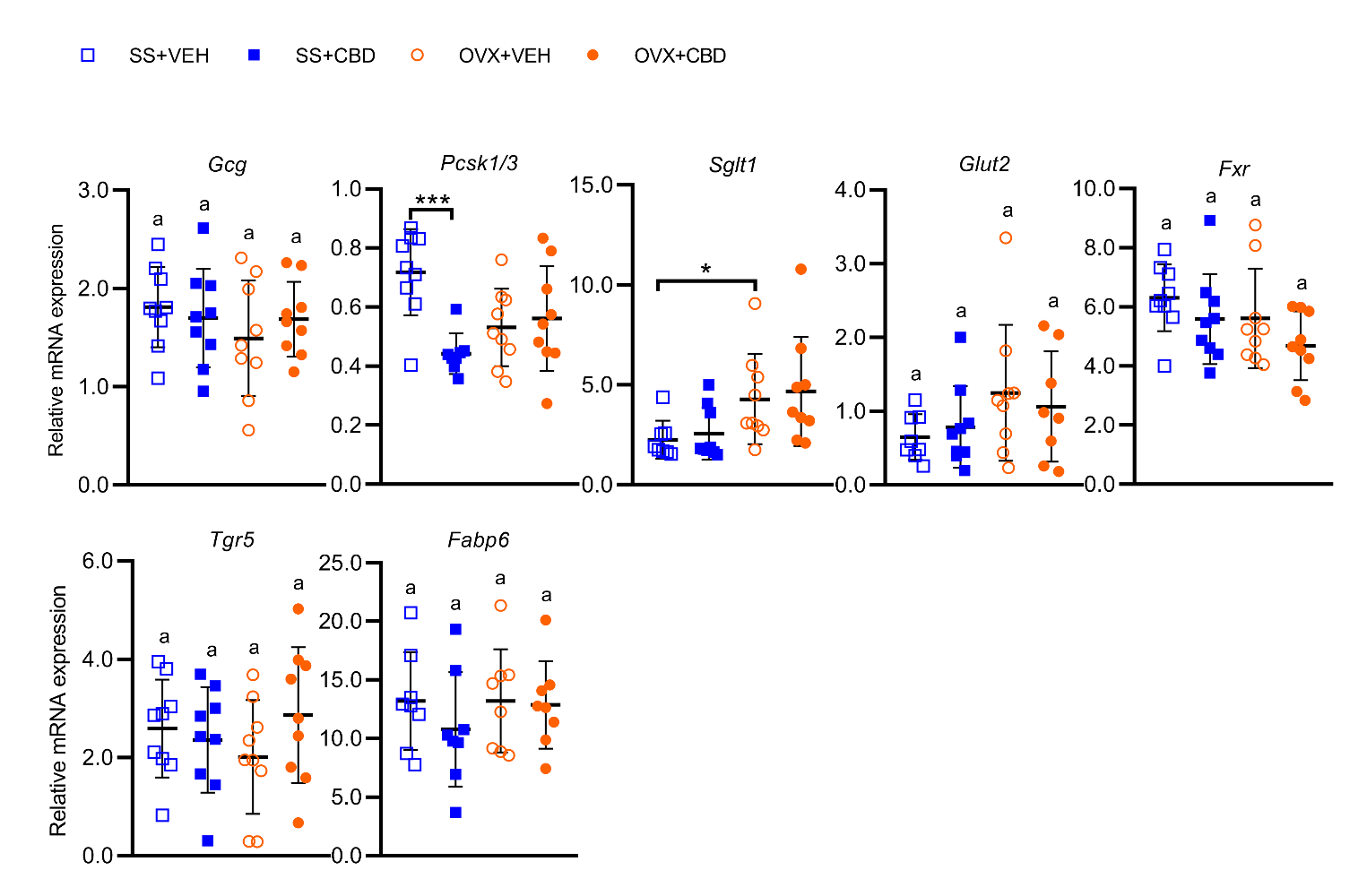
**

**(C)**

**(B)**

**
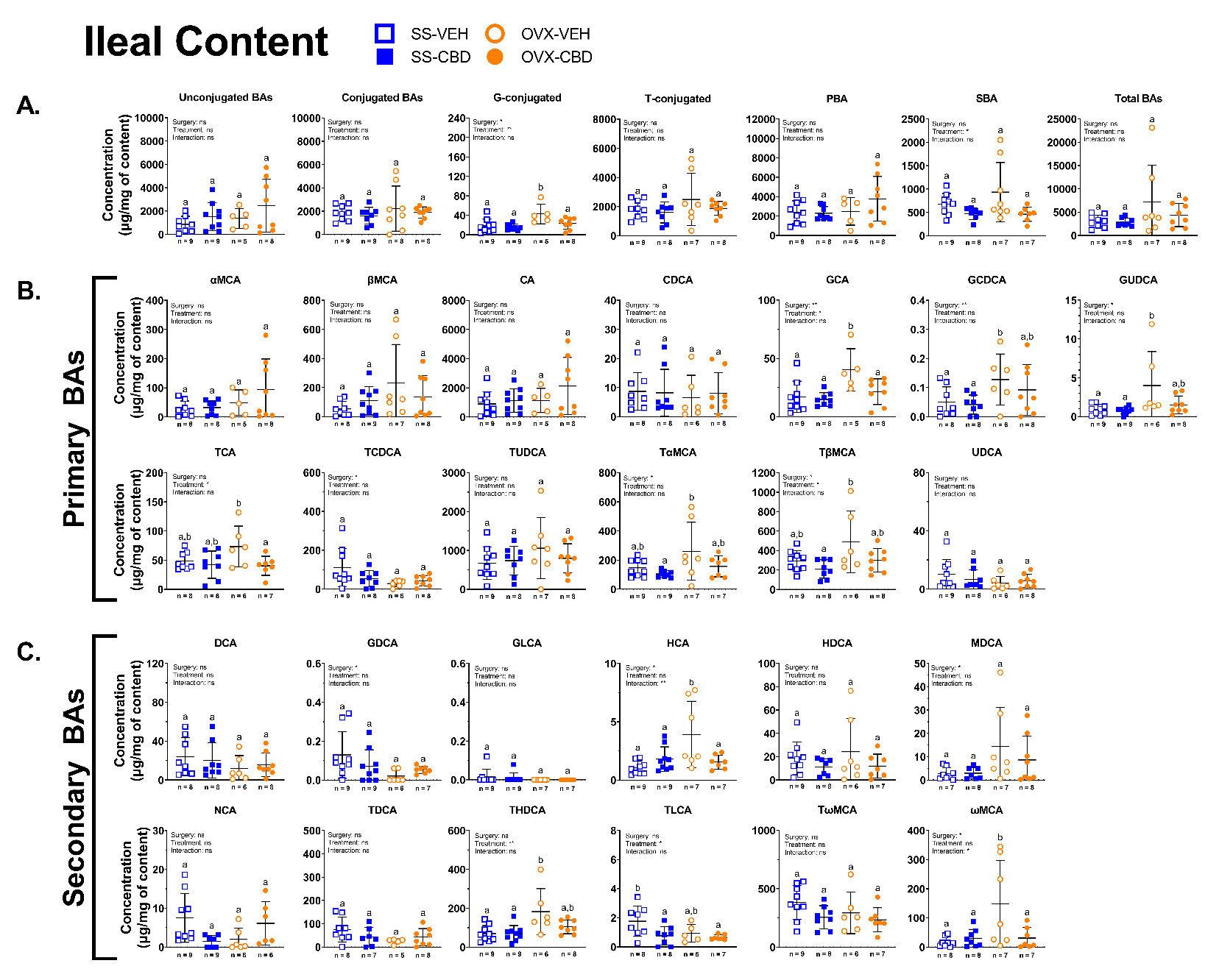
Supplementary Figure 6**

**Supplementary Figure 7**

**
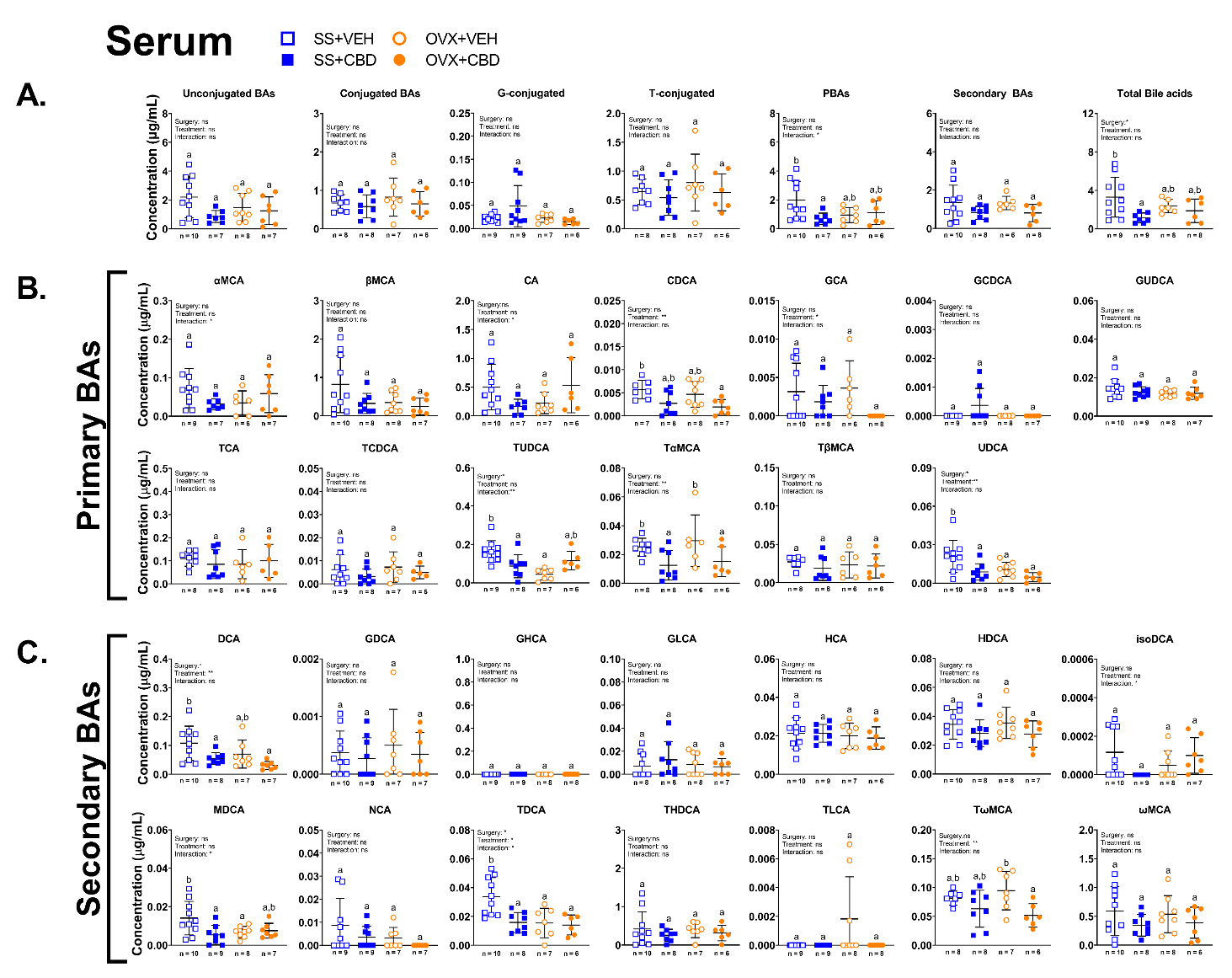
**

**Supplementary Data Figure Legends**

**Supplementary Figure 1. CBD did not affect food intake or OVX-induced changes to body composition or uterine phenotypes**. (**A**) Cumulative food intake. (**B**) Body weights. (**C**) Percentage of fat mass. (**D**) Percentage of lean mass. (**E**) Fat mass (**F**) Lean mass (**G**) Liver weights. (**H**) Liver weight-to-body weight ratio (**I**) Uterine weights, inset photos show representative uterine horns dissected from SS and OVX groups. (**J**) Uterine weight-to-body weight ratio. All data are mean ± SD with n= 9 – 10 mice/ group. Outliers were removed by ROUT test**.** 2-way ANOVA was performed followed by the Benjamini-Hochberg post-hoc test with FDR adjustment, q< 0.05. 2-way ANOVA results for panels A – F are summarized in Supplementary Table 1. Results of post hoc test are summarized in A – F using the pound (#) sign to denote significant difference between SS and OVX groups at indicated time points and in G – J using different letters (a, b) to indicate significant differences between groups.

**Supplemental Figure 2. Spontaneous physical activity of VEH- and CBD-treated SS and OVX groups.** (**A**) X- plane total activity. (**B**) X-plane ambulatory activity. (**C**) Y-plane total activity. (**D**) Y-plane ambulatory activity. (**E**) Z-plane total activity. Data are mean ± SD, n = 9 mice/group. Outliers were removed by ROUT test**.** 2-way ANOVA was performed followed by the Benjamini-Hochberg post-hoc test with FDR adjustment, q< 0.05. 2-way ANOVA results are indicated under each graph. For posthoc test, different letters (a, b) indicate significant differences between groups.

**Supplementary Figure 3. qPCR analyses.** Relative tissue mRNA levels in (**A**) ileum and (**B**) femur. (**C**) OPG/RANKL and RANKL/OPG mRNA ratios. n= 9 mice/group. Outliers were removed by ROUT test**.** Significant differences between groups were determined by two-way ANOVA followed by Holm-Sidak post-hoc test to isolate differences due to surgery or treatment, * p< 0.05, ** p< 0.01, *** p< 0.001.

**Supplementary Figure 4. CBD did not alter cecal SCFAs.** Levels of SCFAs measured in cecal content by GC-MS. Due to required weight of cecal content obtained the following samples were extracted: n= 7 for SS + VEH, n= 6 for SS + CBD, n= 9 for OVX + VEH, n= 7 for OVX + CBD. ROUT test was performed to detect outliers**.** 2-way ANOVA was performed followed by the Benjamini-Hochberg post-hoc test with FDR adjustment, q< 0.05. 2-way ANOVA results are indicated under each graph. * p< 0.05. Posthoc test did not show significant differences between groups.

**Supplementary Figure 5. Diversity metrics of fecal microbial communities and relative abundance of specific genera.** (**A**) Alpha diversity metrics showing differences for Faith’s phylogenetic diversity (Faith PD), Richness, Shannon index, and Pielou’s evenness. Samples extracted were n = 7/ group. Significant difference between groups was determined by Kruskal-Wallis test followed by the Benjamini-Hochberg post-hoc test with FDR adjustment, *q< 0.05, *q<0.01, ***q< 0.001. (**B**) Beta diversity dissimilarity metrics (Bray Curtis, weighted unifrac, unweighted unifrac, and Jaccard) are shown as 2D-principal coordinate analysis (PCoA) plots. Analysis of variance was determined using ADONIS and 10,000X permutation analysis in the vegan package within R Studio v.4.1.2 (R Studio Software, Boston, MA, USA). The r^2^ and p values for surgery and treatment effects are shown. (**C**) Relative abundance of ASVs identified in fecal microbiota (n=7 mice/group; 28 total) were classified at the genera level using a Naïve-Bayes trained Silva taxonomic classifier. ROUT test was performed to detect outliers**.** Within each group, genera with less than 50% prevalence across samples were excluded. Differences were determined by Kruskal-Wallis test followed by the Benjamini-Hochberg post-hoc test with FDR adjustment. For A and C, different letters (a, b, c) indicate significant difference between groups (q< 0.05), while the same letter indicates no difference.

**Supplementary Figure 6. Ileal content bile acid (BA) profile.**Ileal content BAs analyzed in VEH-treated SS (n= 9), CBD-treated SS (n=9), VEH-treated OVX (n=7), and CBD-treated OVX (n=8) mice organized as: (**A**) Grouped BA profiles, (**B**) individual PBAs and (**C**) individual SBAs. Under each graph, n indicates the number of samples per group included in the statistical analysis after removal of outliers as determined by ROUT test (Q=1). Data were tested for normality and significant difference between groups was determined by either Kruskal-Wallis test (for non-normal distribution) or 2-way ANOVA (for normal distribution) followed by the Benjamini-Hochberg post-hoc test with FDR adjustment. The surgery, treatment and interaction results for the 2-way ANOVA test are shown in the upper left corner of each graph and the posthoc test results are indicated by letters (a, b, c) where different letters indicate significant difference between groups (q< 0.05), while the same letter indicates no difference.

**Supplementary Figure 7. Serum BA profile.** Serum BAs analyzed in VEH-treated SS (n= 10), CBD-treated SS (n= 9), VEH-treated OVX (n= 8), and CBD-treated OVX (n= 7) mice organized as: (**A**) Grouped BA profiles, (**B**) individual PBAs and (**C**) individual SBAs. Under each graph, n indicates the number of samples per group included in the statistical analysis after removal of outliers as determined by ROUT test (Q=1). Data were tested for normality and significant difference between groups was determined by either Kruskal-Wallis test (for non-normal distribution) or 2-way ANOVA (for normal distribution) followed by the Benjamini-Hochberg post-hoc test with FDR adjustment. The surgery, treatment and interaction results for the 2-way ANOVA test are shown in the upper left corner of each graph and the posthoc test results are indicated by letters (a, b, c) where different letters indicate significant difference between groups (q< 0.05), while the same letter indicates no difference.

**Supplementary Table 1. ANOVA results for OGTT curve, body weight, fat mass and lean mass**

|  | Time point (min) | 0 | 15 | 30 | 60 | 90 | 120 |  |  |  |
| --- | --- | --- | --- | --- | --- | --- | --- | --- | --- | --- |
| Figure 1 (A) | Surgery | **** | * | **** | *** | *** | ** |  |  |  |
|  | Treatment | ns | ns | ns | * | ns | ns |  |  |  |
|  | Interaction | ns | ns | ns | ns | ns | ns |  |  |  |
|  |  |  |  |  |  |  |  |  |  |  |
|  | Week | 1 | 2 | 3 | 4 | 5 | 6 | 7 | 8 | 9 |
| Supplementary Figure 1 (A) | Surgery | **** | **** | **** | **** | **** | **** | **** | **** | **** |
|  | Treatment | ns | ns | ns | ns | ns | ns | ns | ns | ns |
|  | Interaction | ns | ns | ns | ns | ns | ns | ns | ns | ns |
|  |  |  |  |  |  |  |  |  |  |  |
|  | Week | 10 | 11 | 12 | 13 | 14 | 15 | 16 | 17 | 18 |
|  | Surgery | **** | **** | **** | **** | **** | **** | **** | **** | **** |
|  | Treatment | ns | ns | ns | ns | ns | ns | ns | ns | ns |
|  | Interaction | ns | ns | ns | ns | ns | ns | ns | ns | ns |
|  |  |  |  |  |  |  |  |  |  |  |
|  | Week | 1 | 2 | 3 | 4 | 5 | 6 | 7 | 8 | 9 |
| Supplementary Figure 1 (B) | Surgery | – | – | – | ns | * | * | * | ** | * |
|  | Treatment | – | – | – | ns | ns | ns | ns | ns | ns |
|  | Interaction | – | – | – | ns | ns | ns | ns | ns | ns |
|  |  |  |  |  |  |  |  |  |  |  |
|  | Week | 10 | 11 | 12 | 13 | 14 | 15 | 16 | 17 | 18 |
|  | Surgery | * | * | ** | ** | * | * | ** | ** | ** |
|  | Treatment | ns | ns | ns | ns | ns | ns | ns | ns | ns |
|  | Interaction | ns | ns | ns | ns | ns | ns | ns | ns | ns |
|  |  |  |  |  |  |  |  |  |  |  |
|  | Week | 0 | 4 | 7 | 10 | 14 | 18 |  |  |  |
| Supplementary Figure 1 (C) | Surgery | ** | ** | **** | **** | **** | **** |  |  |  |
|  | Treatment | ns | ns | ns | ns | ns | ns |  |  |  |
|  | Interaction | ns | ns | ns | ns | ns | ns |  |  |  |
|  |  |  |  |  |  |  |  |  |  |  |
|  | Week | 0 | 4 | 7 | 10 | 14 | 18 |  |  |  |
| Supplementary Figure 1 (D) | Surgery | *** | *** | **** | **** | **** | **** |  |  |  |
|  | Treatment | ns | ns | ns | ns | * | ns |  |  |  |
|  | Interaction | ns | ns | ns | ns | ns | ns |  |  |  |
|  |  |  |  |  |  |  |  |  |  |  |
|  | Week | 0 | 4 | 7 | 10 | 14 | 18 |  |  |  |
| Supplementary Figure 1 (E) | Surgery | *** | *** | **** | **** | **** | **** |  |  |  |
|  | Treatment | ns | ns | ns | ns | ns | ns |  |  |  |
|  | Interaction | ns | ns | ns | ns | ns | ns |  |  |  |
|  |  |  |  |  |  |  |  |  |  |  |
|  | Week | 0 | 4 | 7 | 10 | 14 | 18 |  |  |  |
| Supplementary Figure 1 (F) | Surgery | **** | **** | **** | ** | * | **** |  |  |  |
|  | Treatment | ns | ns | ns | ns | ns | ns |  |  |  |
|  | Interaction | * | ns | ns | ns | ns | ns |  |  |  |

**Supplementary Table 2. Taqman assays**

| **Gene name** | **Protein** | **Assay ID** |
| --- | --- | --- |
| *Tnf* | TNFα | Mm00443258_m1 |
| *Il6* | IL6 | Mm00446190_m1 |
| *Il1b* | IL1β | Mm00434228_m1 |
| *Tjp1* | ZO-1 | Mm01320638_m1 |
| *Ocln* | occludin | Mm00500912_m1 |
| *Tnfsf11* | RANKL | Mm00441906_m1 |
| *Tnfrsf11a* | RANK | Mm00437132_m1 |
| *Tnfrsf11b* | OPG | Mm00435454_m1 |
| *Acp5* | TRAP | Mm00475698_m1 |
| *Gpr55* | GPR55 | Mm02621622_s1 |
| *Trpv1* | TRPV1 | Mm01246300_m1 |
| *Nr1h4* | FXR | Mm00436425_m1 |
| *Gpbar1* | TGR5 | Mm04212121_s1 |
| *Nr0b2* | SHP | Mm00442278_m1 |
| *Cnr1* | CB1 | Mm01212171_s1 |
| *Cnr2* | CB2 | Mm02620087_s1 |
| *Fabp6* | FABP6 | Mm00434315_m1 |
| *Pcsk1* | prohormone convertase 1/3 (PC1/3) | Mm00479023_m1 |
| *Gcg* | preproglucagon | Mm00801714_m1 |
| *Slc5a1* | SGLT1 | Mm00451203_m1 |
| *Slc2a2* | GLUT2 | Mm00446229_m1 |
| *Hmbs* | HMBS | Mm01143545_m1 |
| *Gapdh* | GAPDH | Mm99999915_g1 |

| **Supplementary Table 3. Bile acids, cannabidiol, and LC-MS method parameters** | | | | | | | | |
| --- | --- | --- | --- | --- | --- | --- | --- | --- |
| **No.** | **Bile acid or Phytocannabinoid** | **Abbreviation** | **Supplier** | **^1^*m/z***  **[M - H]** | **^2^*m/z***  **[M + H]** | **Cone voltage**  **(CV)** | **Rt (min)** | **IS^3^** |
| 1 | Tauro-ω-muricholic acid | TωMCA | Steraloids | 514.3 | - | 30 | 4.23 | d4-TCA |
| 2 | Glycohyocholic acid | GHCA | Cayman | 464.3 | - | 30 | 4.43 | d4-TCA |
| 3 | Tauro-α-muricholic acid | TαMCA | Steraloids | 514.3 | - | 30 | 4.60 | d4-TCA |
| 4 | Tauro-β-muricholic acid | TβMCA | Steraloids | 514.3 | - | 30 | 5.06 | d4-TCA |
| 5 | Glycocholic acid | GCA | Sigma | 464.3 | - | 30 | 5.64 | d4-TCA |
| 6 | ω-muricholic acid | ωMCA | Steraloids | 407.3 | - | 50 | 5.68 | d4-TCA |
| 7 | Glycoursodeoxycholic acid | GUDCA | Steraloids | - | 414.3/899.7 | 30 | 6.19 | d4-TCA |
| 8 | α-muricholic acid | αMCA | Steraloids | 407.3 | - | 50 | 6.21 | d4-TCA |
| 9 | β-muricholic acid | βMCA | Steraloids | 407.3 | - | 50 | 7.33 | d4-TCA |
| 10 | 3Taurocholic acid-d4 | d4-TCA | Cayman | 518.4 | - | 30 | 7.90 | - |
| 11 | Taurocholic acid | TCA | Steraloids | 514.3 | - | 30 | 8.22 | d4-TCA |
| 12 | Tauroursodeoxycholic acid | TUDCA | Steraloids | 498.3 | - | 30 | 8.54 | d4-TCA |
| 13 | Taurohyodeoxycholic acid | THDCA | Steraloids | - | 464.3 | 30 | 8.56 | d4-TCA |
| 14 | Hyocholic acid (γ-MCA) | HCA | Cayman | - | 355.3 | 30 | 8.60 | d4-TCA |
| 15 | Cholic acid | CA | Sigma | 407.3 | - | 50 | 9.98 | d4-TCA |
| 16 | Murideoxycholic acid | MDCA | Cayman | - | 356.3 | 30 | 10.94 | d4-TCA |
| 17 | Glycochenodeoxycholic acid | GCDCA | Sigma | - | 414.3/899.7 | 30 | 12.13 | d4-TCA |
| 18 | Ursodeoxycholic acid | UDCA | Steraloids | - | 357.3 | 30 | 12.15 | d4-CDCA |
| 19 | Hyodeoxycholic acid | HDCA | Sigma | - | 357.3 | 30 | 12.79 | d4-CDCA |

| 20 | Glycodeoxycholic acid | GDCA | Cayman | - | 414.3/450.3 | 30 | 13.45 | d4-CDCA |
| --- | --- | --- | --- | --- | --- | --- | --- | --- |
| 21 | Nutriacholic acid | NCA | Steraloids | - | 355.2/373.3 | 30 | 15.80 | d4-CDCA |
| 22 | Taurochenodeoxycholic acid | TCDCA | Sigma | - | 464.3 | 30 | 16.06 | d4-CDCA |
| 23 | Taurodeoxycholic acid | TDCA | Sigma | - | 464.3 | 30 | 17.90 | d4-DCA |
| 24 | 3Chenodeoxycholic acid-d4 | d4-CDCA | Cayman | - | 361.3 | 30 | 20.92 | - |
| 25 | Chenodeoxycholic acid | CDCA | Sigma | - | 357.3 | 30 | 21.09 | d4-DCA |
| 26 | 3Deoxycholic acid-d4 | d4-DCA | Steraloids | - | 361.3 | 30 | 22.07 | - |
| 27 | Deoxycholic acid | DCA | Sigma | - | 357.3 | 30 | 22.25 | d4-DCA |
| 28 | Glycolithocholic acid | GLCA | Cayman | - | 416.4/867.7 | 30 | 27.80 | d4-DCA |
| 29 | Isodeoxycholic acid | isoDCA | Cayman | - | 358.3 | 30 | 31.30 | d4-DCA |
| 30 | Taurolithocholic acid | TLCA | Sigma | 482.3 |  | 30 | 33.70 | d4-DCA |
| 31 | 4Cannabidiol | CBD | Bluebird  Botanicals | - | 315.2 | 15 | 38.80 | - |

Detection parameters for 27 BA species, 3 internal standards (IS), and CBD. BAs were injected in ^1^negative and ^2^positive ionization modes. ^3^d4-DCA, d4-CDCA, and d4-TCA were used as ISs. IS calibrant used for each BA was determined based on having similar chemical structure and retention time. ^4^CBD was detected using an external standard curve with no IS calibrant. Selective ion recordings (SIRs) were optimized and used for quantification of each BA species. Base peak(s) were used for quantification of each BA is reported on the table. Peak area (μV*s) was identified using ApexTrek algorithm in Empower 3 software. A smoothing algorithm of 19 was applied to each SIR to resolve peaks. A total of 21 SIRs were established; each corresponding to the reported mass (m/z- or m/z+) of each BA analyzed in serum or ileal content. The same SIR channel was used for BAs with the same ionized isotopic mass. ISs were used to calculate response factors for each BA in samples and standard curves.

| **Supplementary Table 4. Calibration curves, limit of detection, limit of quantification, and coefficient of variance** | | | | | | | |
| --- | --- | --- | --- | --- | --- | --- | --- |
| **No.** | **Bile acid or Phytocannabinoid** | **Abbreviation** | **Calibration Curves^a^** | **R2** | **LOD** | **LOQ** | **CV (%)** |
|  |  |  | y = 1.528(x) + 0.0265 | 0.9990 | 0.00125 | 0.005 |  |
| 1 | Tauro-ω-muricholic acid | TωMCA | y = 1.413(x) + 0.0364 | 0.9993 | 0.001 | 0.006 | 0.13 |
|  |  |  | y = 0.659(x) + 1.9574 | 0.9904 | 0.001 | 0.013 |  |
|  |  |  | y = 0.685(x) + 0.0174 | 0.9959 | 0.0125 | 0.023 |  |
| 2 | Glycohyocholic acid | GHCA | y = 0.739(x) + 0.0466 | 0.9979 | 0.01 | 0.054 | 1.02 |
|  |  |  | y = 1.738(x) + 0.0305 | 0.9988 | 0.01 | 0.058 |  |
|  |  |  | y = 1.078(x) + 2.031 | 0.9955 | 0.001 | 0.0025 |  |
| 3 | Tauro-α-muricholic acid | TαMCA | y = 2.211(x) - 0.021 | 0.9989 | 0.001 | 0.0029 | 0.5 |
|  |  |  | y = 1.887(x) + 0.033 | 0.9998 | 0.001 | 0.005 |  |
|  |  |  | y = 3.548(x) + 0.042 | 0.9987 | 0.001 | 0.002 |  |
| 4 | Tauro-β-muricholic acid | TβMCA | y = 3.046(x) + 0.097 | 0.9992 | 0.001 | 0.002 | 0.34 |
|  |  |  | y = 1.902(x) + 2.645 | 0.9979 | 0.001 | 0.003 |  |
|  |  |  | y = 0.964(x) - 0.0474 | 0.9990 | 0.01 | 0.008 |  |
| 5 | Glycocholic acid | GCA | y = 0.425(x) - 0.0002 | 1.0 | 0.01 | 0.02 | 0.5 |
|  |  |  | y = 0.1184x - 0.0028 | 0.9998 | 0.01 | 0.04 |  |
|  |  |  | y = 0.0535(x) - 0.0046 | 0.9881 | 0.0125 | 0.06 |  |
| 6 | ω-Muricholic acid | ωMCA | y = 0.4378(x) + 0.0122 | 0.9991 | 0.01 | 0.06 | 0.32 |
|  |  |  | y = 0.1545(x) - 0.0005 | 0.9986 | 0.01 | 0.2 |  |
|  |  |  | y = 9.640(x) + 0.1554 | 0.9993 | 0.00125 | 0.003 |  |
| 7 | Glycoursodeoxycholic acid | GUDCA | y = 9.346(x) + 0.0835 | 1.0 | 0.001 | 0.004 | 0.91 |
|  |  |  | y = 1.413(x) + 0.0378 | 0.9994 | 0.001 | 0.023 |  |
|  |  |  | y = 0.307(x) - 0.0036 | 0.9982 | 0.0125 | 0.113 |  |
| 8 | α-Muricholic acid | αMCA | y = 0.136(x) - 0.0113 | 0.9927 | 0.01 | 0.078 | 0.8 |
|  |  |  | y = 0.604(x) + 0.0129 | 0.9993 | 0.01 | 0.15 |  |
|  |  |  | y = 0.224(x) - 0.0044 | 0.9992 | 0.0125 | 0.02 |  |
| 9 | β-Muricholic acid | βMCA | y = 0.078(x) - 0.0082 | 0.9916 | 0.01 | 0.025 | 0.5 |
|  |  |  | y = 0.511(x) + 0.0192 | 0.9988 | 0.01 | 0.059 |  |
|  |  |  | y = 2.9485x + 10.022 | 0.9939 | 0.0125 | 0.02 |  |
| 10 | Taurohyodeoxycholic acid | THDCA | y = 10.55(x) + 0.3471 | 0.9999 | 0.01 | 0.02 | 0.6 |
|  |  |  | y = 8.4552x + 0.3572 | 0.9987 | 0.01 | 0.006 |  |
|  |  |  | y = 0.229(x) + 0.0284 | 0.9981 | 0.00125 | 0.02 |  |
| 11 | Tauroursodeoxycholic acid | TUDCA | y = 0.061(x) + 0.1139 | 0.9973 | 0.001 | 0.025 | 0.4 |
|  |  |  | y = 0.2874x + 0.0008 | 0.9987 | 0.001 | 0.08 |  |

| 12 | Taurocholic acid | TCA | y = 1.172(x) + 1.0003 y = 1.779(x) + 0.0358 y = 1.776(x) - 0.0442 | 0.9992  0.9913  0.9994 | 0.00125  0.001  0.001 | 0.002  0.002  0.003 | 0.31 |
| --- | --- | --- | --- | --- | --- | --- | --- |
| 13 | Hyocholic acid (γ-MCA) | HCA | y = 5.963 (x) + 0.214  y = 6.753 (x) + 0.076  y = 2.644 (x) + 0.063 | 0.9999  0.9997  0.9975 | 0.00125  0.001  0.001 | 0.003  0.004  0.009 | 2.8 |
| 14 | Cholic acid | CA | y = 0.595(x) + 0.0206 y = 0.437(x) - 0.0522 y = 0.357(x) + 0.1998 | 0.9991  0.9965  0.9913 | 0.00125  0.001  0.001 | 0.031  0.023  0.04 | 0.25 |
| 15 | Taurochenodeoxycholic acid | TCDCA | y = 1.127(x) - 0.0198 y = 1.383(x) - 0.0221 y = 1.332(x) - 0.0064 | 0.9999  0.9998  0.9998 | 0.0125  0.01  0.01 | 0.041  0.042  0.051 | 0.17 |
| 16 | Ursodeoxycholic acid | UDCA | y = 1.091(x) + 0.0195 y = 1.046(x) + 0.0042  y = 0.922(x) + 0.0112 | 0.9996  0.9999  0.9999 | 0.00125  0.001  0.001 | 0.008  0.009  0.008 | 0.51 |
| 17 | Glycochenodeoxycholic acid | GCDCA | y = 5.434(x) - 0.192 y = 14.70(x) + 0.318 y = 9.241(x) + 0.268 | 0.9947  0.9998  0.9992 | 0.0125  0.001  0.001 | 0.005  0.008  0.014 | 0.59 |
| 18 | Hyodeoxycholic acid | HDCA | y = 1.251(x) - 0.018  y = 1.208(x) + 0.034 y= 0.949(x) + 0.022 | 0.9998  0.9991  0.9996 | 0.0125  0.01  0.01 | 0.068  0.071  0.09 | 0.16 |
| 19 | Glycodeoxycholic acid | GDCA | y = 23.08(x) + 0.1789 y = 10.07(x) + 0.4875 y = 2.431(x) + 0.0295 | 0.9990  0.9980  0.9995 | 0.0125  0.01  0.01 | 0.034  0.079  0.024 | 0.65 |
| 20 | Nutriacholic acid | NCA | y = 0.2775(x) - 0.0032 y = 0.4863(x) + 0.0171 y = 0.1649(x) + 0.0067 | 0.9987  0.9992  0.9997 | 0.125  0.1  0.1 | 0.26  0.23  0.38 | 1.55 |
| 21 | Taurodeoxycholic acid | TDCA | y = 1.671(x) - 0.0022  y = 1.685(x) - 0.0225 y = 1.286(x) - 0.0578 | 0.9993  0.9998  0.9983 | 0.0125  0.01  0.01 | 0.021  0.021  0.027 | 1.9 |
| 22 | Murideoxycholic acid | MDCA | y = 3.610(x) + 0.1912 y = 3.933(x) + 0.2862 y = 2.167(x) + 0.0274 | 0.9981  0.9962  0.9986 | 0.0125  0.01  0.01 | 0.03  0.012  0.012 | 2.28 |
| 23 | Isodeoxycholic acid | isoDCA | y = 3.638(x) - 0.2512 y = 9.422(x) + 0.3299 y = 9.533(x) - 0.0057 | 0.9994  0.9990  1.0 | 0.00125  0.001  0.001 | 0.006  0.005  0.002 | 0.08 |
| 24 | Chenodeoxycholic acid | CDCA | y = 1.239(x) + 0.0262  y = 1.633(x) - 0.0309 y = 1.698(x) + 0.0473 | 0.9995  0.9999  0.9945 | 0.0125  0.01  0.01 | 0.054  0.052  0.072 | 0.26 |

| 25 | Deoxycholic acid | DCA | y = 1.234(x) - 0.0016 y = 1.345(x) + 0.0012 y = 0.946(x) + 0.0402 | 0.9999  1.0  0.9990 | 0.00125  0.001  0.001 | 0.004  0.004  0.005 | 0.25 |
| --- | --- | --- | --- | --- | --- | --- | --- |
| 26 | Glycolithocholic acid | GLCA | y = 2.126(x) + 0.0538 y = 2.511(x) + 0.0836  y = 3.206(x) - 0.0242 | 0.9993  0.9992  1.0 | 0.0125  0.01  0.01 | 0.08  0.05  0.08 | 1.69 |
| 27 | Taurolithocholic acid | TLCA | y = 1.129(x) - 0.062 y = 1.619(x) + 0.045 y = 1.833(x) - 0.055 | 0.9971  0.9992  0.9996 | 0.0125  0.01  0.01 | 0.067  0.059  0.096 | 2.2 |
| 28 | ^e^Cannabidiol | CBD | y = 5E+06x – 310850 y = 8E+06x – 280526 y = 3E+06x + 204418 | 0.9987  0.9994  0.9930 | 0.0125  0.01  0.01 | 0.025  0.02  0.02 | 1.02 |

BAs were quantified against a 7- to 10-point external standard curve (0.001μg/mL to 200 μg/mL) including 27 pure external BA standards injected in duplicate at the beginning, middle, and/or end of each sample set run to generate 3 calibration curves. Linear calibration curves were generated using external BA standards calibrated with deuterated internal standards (1 μg/mL) to calculate response factors for each BA species in ileal content and serum samples. BA species are listed by common name and respective abbreviation.

^a^Peak area (μV*s) of each BA standard concentration (μg/mL) was averaged to generate a linear calibration curve for each sample set. Serum or ileal bile acids for a given sample set were quantified against the assigned beginning, middle, and/or end calibration curve. R^2^ values for each calibration curve. BA concentrations in serum are shown as μg/mL and in ileal content as μg/mg.

^b^Coefficient of variance (CV) for each BA was determined by comparing mean and standard deviation of peak areas (μV*s) for quality control of samples and BA standards.

^c^Limit of detection (LOD) for each BA species was determined by the lowest quantifiable peak discernible via manual integration with a signal to noise (S/N) ratio greater than 2 for each single ion recording (SIR). LOD values correspond to BA standards detected in both injections for each standard curve point.

^d^Limit of quantification (LOQ) for each linear calibration curve was determined using the equation (10*σ/s), for which ‘σ’ is the standard deviation of the response factor for each BA standard curve, and ‘s’ is the slope of the y-intercept for each linear calibration curve.

^e^CBD concentration (μg/mL) was determined using a linear external standard curve of CBD isolate injected and pre-screened for contaminating peaks or other cannabinoid compounds.

**Supplemental Methods**

*Chemicals and materials*

Experiments were performed using Optima LC-MS grade formic acid, methanol, acetonitrile, and water purchased from Fisher Scientific (Hampton, NH, USA). Oasis Prime HLB 1cc Cartridges (30 mg) were purchased from Waters (Milford, MA, USA) for filtration of phospholipids from serum samples. Corning Costar Spin-X centrifuge tubes with 0.22μm nylon membrane (Corning, NY, USA), were used to filter all bile acid (BA) extracts prior to injecting. As shown in **Supplementary Table 3 online**; Cannabidiol isolate was purchased from Bluebird Botanicals (Louisville, CO, USA), 12 unconjugated, 9 taurine conjugated, and 6 glycine conjugated BAs were ordered from either Sigma-Aldrich Inc (St. Louis, MO, USA), Cayman Chemical (Ann Arbor, MI, USA), or Steraloids Inc. (Newport, RI, USA). The internal standards (ISs) taurocholic acid-d4 (TCA-d4), chenodeoxycholic acid-d4 (CDCA-d4), and deoxycholic acid-d4 (DCA-d4) were purchased from Cayman Chemicals.

**Bile acid analysis**

*Preparation of standard curves,*

Stock solutions (0.5 mg/mL - 1 mg/mL) of 27 individual BAs were prepared and stored at -20°C. Conjugated BAs were dissolved in 50% methanol while unconjugated BAs were prepared in 100% methanol for stability. An IS solution (1 μg/mL each) containing a mixture of TCA-d4, CDCA-d4, and DCA-d4, was prepared in 50% methanol. Deuterated BA were used as ISs to correct for variability in recovery of BA species. Using stock solutions, 27 BAs were diluted and pooled such that each was present at a concentration of 5 µg/mL (50% methanol) in a final BA mixture. The BA mixture was further diluted with 50% methanol to give final concentrations of 0.00125 μg/mL, 0.0025 μg/mL, 0.0125 μg/mL, 0.125 μg/mL, 1.25 μg/mL, 2.5 μg/mL, 5 μg/mL (7-point standard curve).

Tauro-omega muricholic acid, taurohyodeoxycholic acid, tauroursodeoxycholic acid, tauro-alpha muricholic acid, tauro-beta muricholic acid, taurocholic acid, and cholic acid were at concentrations above 5 μg/mL in ileal content therefore to quantify these 7 BAs, standards of higher concentration were prepared and pooled to give final concentrations of 25, 50, and 200 μg/mL. For preparation of the calibration curves, 200 μL of IS solution (1 µg/mL) was placed into microfuge tubes and dried using a CentriVap concentrator (LABCONCO) coupled with a CentriVap Cold Trap (LABCONCO). Subsequently, 200 μL of each concentration of BA mix was added to the tube containing the dried ISs prior to injection. Serum BAs were quantified against a 7-point standard curve. Ileal content BAs were quantified against a 7-point stand curve except for BAs mentioned above that were outside this range; these were quantified against a 10-point stand curve.

*Preparation of serum samples*

Serum samples (n = 7 - 10/group) were prepared as previously described (Tveter *et al.,* 2020. BMJ Open Diabetes Res Care Aug;8(1):e001386.) with some modifications. In brief, aliquots of the IS (200 μL) were placed into individual microfuge tubes and dried using CentriVap concentrator. Ice cold acetonitrile (150 μL) was added to 50 μL of each serum samples and incubated at – 20 °C for 1 hr to precipitate proteins. Samples were centrifuged at 4 °C for 10 min at 16,000 *g* and supernatants (~ 200 μL) were added to HLB Oasis Prime cartridges (1 cc, 30 mg sorbent, Waters Corp.) and allowed to sorb for 5 min. The column was washed with 1 mL of 5% acetonitrile and the wash fraction was collected in a separate microfuge tube. 1 mL of 90% acetonitrile was added to cartridge and the eluent was collected in a second microfuge tube. The wash and eluent were dried separately using CentriVap concentrator (LABCONCO) coupled with CentriVap Cold Trap (LABCONCO).

Each wash and eluent was reconstituted separately in 100 μL of 50% methanol and then injected sequentially for each sample. To prevent cross contamination of water-soluble compounds, eluent fractions (BA-predominant faction) were injected first followed by the wash fractions, which contained lower concentrations of BAs. A set of standards (n = 8 including the blank) and QC sample (BA mix of 5, 2.5 or 1.25 μg/mL) were analyzed once at the beginning of the run and after every 15 samples injected to obtain a standard curve for each sample set. Details of standard curves are in **Supplementary Table 4**.

*Preparation of ileal content samples*

Individual ileal content samples (n= 6 - 9 /group) were collected into microfuge tubes by flushing lumen of the ileal segment (~5 cm from distal end) with 1x PBS (pH 7.4). The ileal content was freeze-dried (LABCONCO) overnight to evaporate PBS and dry weight (mg) of ileal content was recorded. To subtract weight contributed by salts in PBS, 3 tubes containing 1 mL of 1x PBS were freeze-dried and their mean weight was subtracted from dried ileal content weights. Samples with ileal content less than 1 mg after subtracting average PBS weight were excluded from the analysis (n=2). 600 μL of 90% acetonitrile/ 9.9% water/ 0.1% formic acid (v/v/v) was added to dry ileal content, vortexed for 1 min, and left on a benchtop shaker at 4°C for 1 hour. After extraction, samples were centrifuged at 15 000 *g* for 10 min and the supernatant was collected in a clean microfuge tube. For the second round of extraction, 600 μL of 80% methanol/ 20% water (v/v) was added to the pellet, vortexed for 2 min, and extracted as described above. Supernatants were pooled and solvent was evaporated in speed vacuum (CentriVap concentration system with cold trap, Labconco, Kansas City, MO USA) and resuspended in 200 μL of 80% methanol. Samples were diluted 50x using 80% methanol prior to injection. Percent recovery of deuterated ISs was determined for each sample.

For both serum and ileal content samples, the percent recovery of deuterated ISs, limit of detection (LOD), limit of quantification (LOQ), and coefficient of variance (CV) are presented in **Supplementary Table 4**. Recoveries ranged 74 - 115% for TCA-d4, 92 - 126% for DCA-d4, and

96% - 119% for CDCA-d4.

*LC-MS of BAs and CBD in serum and ileal content*

Data was generated using an Alliance e2695 HPLC system coupled to a 2998 Photodiode array detector and an Acquity QDa detector mass spectrometer equipped with an electrospray interphase (ESI, Waters Milford, MA, USA), an autosampler, and a Vacuubrand pump (Essex, CT). For each sample, technical duplicates were injected (10 μL) and the following instrument and processing methods were applied:

The HPLC column was held at 40 °C during separation. Analytes were separated on a Cortecs C18+ column (4.6 x 150 mm and 2.7 μm particle size, Waters Milford, MA, USA). HPLC mobile phases used for the gradient conditions consisted of 0.1% formic acid in acetonitrile (Solution A) and 0.1% formic acid in water (Solution B).

While maintaining a constant flow rate of 1 mL/min, the analytes were eluted using the following gradient: a linear gradient from 35% to 50% A over 30 min, the gradient was then held constant at 50% A for 1 min, followed by an immediate transition to 65% A for 9 min (until 40^th^ min) followed by a gradual increase to 90% A over 2 min, then held at held at 90% A for 6 min (until 48^th^ min). The washout gradient started at 90% A and transitioned to 10% A from 48 - 54 min before returning to initial conditions (35% A) at 54.1 min. The column was allowed to equilibrate for 6 min in 35% A (until 60 min) before the next injection.

The instrument settings for the QDa MS detector included total ion chromatograms (TIC) and 21 single ion recording (SIR) channels operating simultaneously in positive and negative (ES +/-) full scan modes with scan range of 50 - 1200 mass/charge (m/z) ratio using centroid data collection. Multiple cone voltages (CV) of 15, 30, and 50 were simultaneously utilized to obtain optimal ionization of each BA species. In advanced ionization mode the mass (m/z) of the base peaks (i.e., SIRs) in **Supplementary Table 3** were added to the instrument method in Empower software prior to injecting samples. The following electrospray interphase settings were used: Gain 1, 600°C probe, source temperature of 120°C, and capillary voltage positive 1.5 kV and negative

0.8 kV. LC-MS data was acquired using Empower 3 V.1 software (Waters Milford, MA, USA).

Prior to sample analysis, a processing method was generated by injecting individual BAs (0.5 - 1 mg/mL). Each BA’s retention time, retention window, and peak area (μV*sec) was recorded and used to determine IS reference and detect BA species.

For the BA standards, one calibration curve for each BA was generated by averaging the peak areas of duplicate injections. The corresponding IS reference for each BA species was used to create a standard curve for the response factor (i.e., ratio of external standard to internal standard) used to calculate the concentrations (μg/mL) of serum and ileal content bile acids (**Table 1**). CBD was quantified based on an external standard only using above instrument conditions.

**Gut microbiota analysis**

*QIIME analysis*

Paired-end sequences were imported into QIIME2-2021.4 (Bolyen et al., 2019), demultiplexed, and quality filtered using the q2‐demux plugin. Demultiplexed sequences were denoised, decluttered, and merged using q2-dada2 (Callahan et al., 2016) to generate amplicon sequence variants (ASVs). Ten base pairs were trimmed from 5’ end of forward and reverse reads. Forward reads were not truncated at 3’ end but reverse reads were truncated to include 233 bp. 80-90% of all sequences were merged. Unique features (i.e., ASVs) were aligned with mafft (Katoh et al., 2002) using q2‐alignment and used to construct a phylogeny with fasttree2 (Price et al., 2010) using q2‐phylogeny. Sequences were rarified to 70,250 sequences per samples. Alpha and beta diversity metrics (Faith, 1992, Lozupone and Knight, 2005, Lozupone et al., 2007) and Principle Coordinate Analysis (PCoA) were estimated using q2‐diversity. Taxonomy was assigned to ASVs using the q2‐feature‐classifier (Bokulich et al., 2018) and classify‐sklearn Naïve Bayes taxonomy classifier trained on the Silva 138 OTUs full-length reference sequences. Difference between relative abundance of ASVs classified at the phylum and genera-level were analyzed. An analysis of variance using distance matrices (Bray Curtis, Jaccard, Unweighted and Weighted UniFrac) were individually determined using ADONIS and 10,000X permutation analysis in the vegan package within R Studio V.4.1.2 (R Studio Software, Boston, Massachusetts, USA). Exact q and/or p values are indicated for notable trends.

**References**

BOKULICH, N. A., KAEHLER, B. D., RIDEOUT, J. R., DILLON, M., BOLYEN, E., KNIGHT, R., HUTTLEY, G. A. & CAPORASO, J. G. 2018. Optimizing taxonomic classification of marker-gene amplicon sequences with QIIME 2’s q2-feature-classifier plugin. *Microbiome,* 6**,** 1-17.

BOLYEN, E., RIDEOUT, J. R., DILLON, M. R., BOKULICH, N. A., ABNET, C. C., AL-GHALITH, G. A., ALEXANDER, H., ALM, E. J., ARUMUGAM, M., ASNICAR, F., BAI, Y., BISANZ, J. E., BITTINGER, K., BREJNROD, A., BRISLAWN, C. J., BROWN, C. T., CALLAHAN, B. J., CARABALLO-RODRÍGUEZ, A. M., CHASE, J., COPE, E. K., DA SILVA, R., DIENER, C., DORRESTEIN, P. C., DOUGLAS, G. M., DURALL, D. M., DUVALLET, C., EDWARDSON, C. F., ERNST, M., ESTAKI, M., FOUQUIER, J., GAUGLITZ, J. M., GIBBONS, S. M., GIBSON, D. L., GONZALEZ, A., GORLICK, K., GUO, J., HILLMANN, B., HOLMES, S., HOLSTE, H., HUTTENHOWER, C., HUTTLEY, G. A., JANSSEN, S., JARMUSCH, A. K., JIANG, L., KAEHLER, B. D., KANG, K. B., KEEFE, C. R., KEIM, P., KELLEY, S. T., KNIGHTS, D., KOESTER, I., KOSCIOLEK, T., KREPS, J., LANGILLE, M. G. I., LEE, J., LEY, R., LIU, Y.-X., LOFTFIELD, E., LOZUPONE, C., MAHER, M., MAROTZ, C., MARTIN, B. D., MCDONALD, D., MCIVER, L. J., MELNIK, A. V., METCALF, J. L., MORGAN, S. C., MORTON, J. T., NAIMEY, A. T., NAVAS-MOLINA, J. A., NOTHIAS, L. F., ORCHANIAN, S. B., PEARSON, T., PEOPLES, S. L., PETRAS, D., PREUSS, M. L., PRUESSE, E., RASMUSSEN, L. B., RIVERS, A., ROBESON, M. S., ROSENTHAL, P., SEGATA, N., SHAFFER, M., SHIFFER, A., SINHA, R., SONG, S. J., SPEAR, J. R., SWAFFORD, A. D., THOMPSON, L. R., TORRES, P. J., TRINH, P., TRIPATHI, A., TURNBAUGH, P. J., UL-HASAN, S., VAN DER HOOFT, J. J. J., VARGAS, F., VÁZQUEZ-BAEZA, Y., VOGTMANN, E., VON HIPPEL, M., WALTERS, W., et al. 2019. Reproducible, interactive, scalable and extensible microbiome data science using QIIME 2. *Nature Biotechnology,* 37**,** 852-857.

CALLAHAN, B. J., MCMURDIE, P. J., ROSEN, M. J., HAN, A. W., JOHNSON, A. J. A. & HOLMES, S. P. 2016. DADA2: High-resolution sample inference from Illumina amplicon data. *Nature methods,* 13**,** 581-583.

FAITH, D. P. 1992. Conservation evaluation and phylogenetic diversity. *Biological conservation,* 61**,** 1-10.

KATOH, K., MISAWA, K., KUMA, K. I. & MIYATA, T. 2002. MAFFT: a novel method for rapid multiple sequence alignment based on fast Fourier transform. *Nucleic acids research,* 30**,** 3059-3066.

LOZUPONE, C. & KNIGHT, R. 2005. UniFrac: a new phylogenetic method for comparing microbial communities. *Applied and environmental microbiology,* 71**,** 8228-8235.

LOZUPONE, C. A., HAMADY, M., KELLEY, S. T. & KNIGHT, R. 2007. Quantitative and qualitative β diversity measures lead to different insights into factors that structure microbial communities. *Applied and environmental microbiology,* 73**,** 1576-1585.

PRICE, M. N., DEHAL, P. S. & ARKIN, A. P. 2010. FastTree 2–approximately maximum-likelihood trees for large alignments. *PloS one,* 5**,** e9490.
